# Proteomic analysis of central amygdala systems regulated by mifepristone in the context of alcohol dependence

**DOI:** 10.1016/j.neuropharm.2025.110728

**Published:** 2025-10-21

**Authors:** Stephanie S. Lee, Laura Monteagudo Romero, John W. Lammons, Jennifer M. Klein, Kimberly N. Edwards, Jessie J. Guidry, Scott Edwards

**Affiliations:** a Department of Physiology, Louisiana State University Health Sciences Center, New Orleans, LA, 70112, USA; b Department of Microbiology, Immunology, and Parasitology, Louisiana State University Health Sciences Center, New Orleans, LA, 70112, USA; c Department of Biochemistry and Molecular Biology and Proteomics Core Facility, Louisiana State University Health Sciences Center, New Orleans, LA, 70112, USA; d Alcohol and Drug Abuse Center of Excellence, Louisiana State University Health Sciences Center, New Orleans, LA, 70112, USA; e Comprehensive Alcohol – HIV/AIDS Research Center, Louisiana State University Health Sciences Center, New Orleans, LA, 70112, USA

**Keywords:** Alcohol dependence, Central amygdala, Glucocorticoid, Mifepristone, Proteomics

## Abstract

Alcohol use disorder (AUD) is a psychiatric disorder characterized by escalated alcohol use and the emergence of negative affective symptoms. Studies have described a dysregulation of glucocorticoid receptor (GR) signaling in the context of AUD at the levels of the hypothalamic-pituitary-adrenal (HPA) axis and central amygdala (CeA). A functional increase in CeA GR activity occurs during alcohol withdrawal in alcohol-dependent animal models, and the GR antagonist mifepristone reduces alcohol-seeking and drinking behaviors in both rodents and humans. The aims of this study were to determine differential CeA protein expression in alcohol-dependent rats and examine the effects of mifepristone treatment in this context. Male and female Wistar rats were exposed to chronic intermittent ethanol vapor (CIEV) or air (control) for 10 weeks. In week 7, half of the animals in each group received either subcutaneous placebo pellets or mifepristone pellets. At the end of Week 10, the rats were **euthanized** during acute CIEV withdrawal (and identical time in control groups). The CeA was analyzed using discovery-based proteomics and Ingenuity Pathway Analysis (IPA). In males, among the 3050 proteins detected, 274 proteins were significantly altered by alcohol, and 158 of these proteins were normalized by mifepristone. In females, among the 2631 detected proteins, 37 proteins were altered by alcohol, and 13 of these proteins were normalized by mifepristone. Alcohol and mifepristone elicited sex-dependent alterations in protein expression within the CeA, confirming and extending previous research to highlight specific proteins and pathways as medication targets to offer novel therapeutic avenues for treating AUD.

## 1. Introduction

Alcohol use disorder (AUD) is a debilitating psychiatric disorder characterized by an uncontrollable escalation of alcohol consumption despite the concurrent emergence of adverse social, occupational, or health-related consequences. In the United States, over 63 % of individuals aged 12 or older have reported alcohol use, with AUD affecting more than 10 % of the population in 2022 (“2022 NSDUH Annual National Report | CBHSQ Data,” 2023). The ramifications of AUD transcend the boundaries of mere excessive drinking, encompassing a multifaceted interplay of neurobiological factors, including the emergence of negative emotional states during withdrawal. Despite its pervasive impact on individuals worldwide, the efficacy of established psychotherapeutic and pharmacotherapeutic interventions for AUD remains limited, highlighting the pressing need for developing more efficacious therapeutic strategies for both men and women (Koob et al., 2009; Ray et al., 2021).

The central amygdala (CeA) stands as a pivotal locus wherein aversive stimuli converge to generate emotional responses (Gallagher and Chiba, 1996; Koob, 2008; LeDoux, 2007). It has garnered attention for its implication in anxiety, stress-related disorders, and the reinforcing effects of alcohol and other substances of abuse (Koob, 2008; Roberto et al., 2021). The CeA resides within a broader conceptual macrostructure in the basal forebrain known as the extended amygdala, comprising the CeA itself, the nucleus accumbens shell (NAc), and the lateral segment of the bed nucleus of the stria terminalis (BNST) (Heimer et al.,. Interactions across the CeA and the rest of the extended amygdala play a critical role in stress sensitization and the transition from limited, recreational substance use to dependence (Egli et al., 2012). Emerging evidence highlights the CeA’s involvement in the negative emotional states that frequently underlie excessive alcohol consumption in individuals with AUD (Gilpin et al., 2015; Roberto et al., 2021).

Previous research has revealed a dysregulation of glucocorticoid signaling in AUD, manifesting both at the level of the hypothalamic-pituitary-adrenal (HPA) axis (Blaine and Sinha, 2017; Stephens and Wand, 2012) and within the CeA (Edwards et al., 2015; Gilpin et al., 2015). The excessive activation of brain glucocorticoid receptors (GRs) is posited to facilitate cognitive/executive dysfunction, negative emotional states, and escalated alcohol consumption, all of which are common features of AUD (Béracochéa et al., 2019; Blaine and Sinha, 2017; Edwards et al., 2015). Specifically, studies have demonstrated increased GR activity within the CeA during alcohol withdrawal in animal models of dependence. Additionally, genetically selected Marchi-gian Sardinian alcohol-preferring (msP) rats exhibit heightened alcohol preference and increased anxiety-like behavior in comparison to their non-alcohol-preferring Wistar counterparts, concomitant with elevated phosphorylated GR levels in the CeA of male msP rats (Natividad et al., 2021). Furthermore, individuals with a history of opioid dependence have displayed a significant upregulation of GR pathway genes within the CeA compared to individuals without such a history (Carmack et al., 2022), suggesting that GR dysregulation may extend to other misused substances.

From a functional standpoint, the GR antagonist mifepristone has been shown to reduce alcohol consumption and mitigate craving symptoms in both rodent models of alcohol dependence and in humans suffering from AUD (Béracochéa et al., 2019; Jacquot et al., 2008; Vendruscolo et al., 2015), although its impact on innate anxiety-like behaviors is negligible (Vozella et al., 2021). At the cellular level, GR signaling exerts both genomic and non-genomic effects across all organ systems including the brain. Genomic signaling occurs via GR translocation to the nucleus upon glucocorticoid binding, where GR associates with glucocorticoid response elements (GRE) to regulate transcription of stress- and plasticity-related genes, such as brain-derived neurotrophic factor (BDNF), FK506 binding protein 5 (FKBP5), and serum/glucocorticoid-regulated kinase 1 (SGK1) (Anacker et al., 2013; Malekpour et al., 2023; Suri and Vaidya, 2013). In parallel, non-genomic mechanisms are believed to be associated with plasma membrane-bound GRs to rapidly activate intracellular signaling cascades such as ERK/MAPK, PI3K/AKT, and CaMKII/CREB pathways, modulating synaptic plasticity, cellular excitability, and emotional regulation (Chen et al., 2012; Evanson et al., 2010; Groeneweg et al., 2011; Langer et al., 2025; Panettieri et al., 2019). Alterations in these pathways likely further contributes to additional genomic dysregulation and represent plausible mechanisms through which chronic mifepristone treatment could exert profound and widespread regulatory effects on CeA neurobiology. Nevertheless, substantial gaps persist in our comprehension of the underlying neurobiological mechanisms governing behavioral alterations in the context of AUD and mifepristone treatment, while elucidation of sex-specific markers of chronic alcohol use and mifepristone efficacy necessitates further research.

Given the intricate interplay observed between the CeA, GR signaling, and AUD, this study investigated their interaction. The primary objectives of this investigation include 1) identification of differential protein expression patterns within the CeA of alcohol-dependent male and female Wistar rats and 2) assessment of mifepristone’s influence on these neurobiological alterations. By illuminating sex-dependent changes in alcohol-driven protein expression profiles and their potential as targets for intervention, this research aimed to advance our comprehension of the neurobiological underpinnings of AUD and open avenues for novel therapeutic approaches for AUD and stress-related co-morbid conditions.

## 2. Materials and methods

### 2.1. Animal experiments

**Fig. 1.**
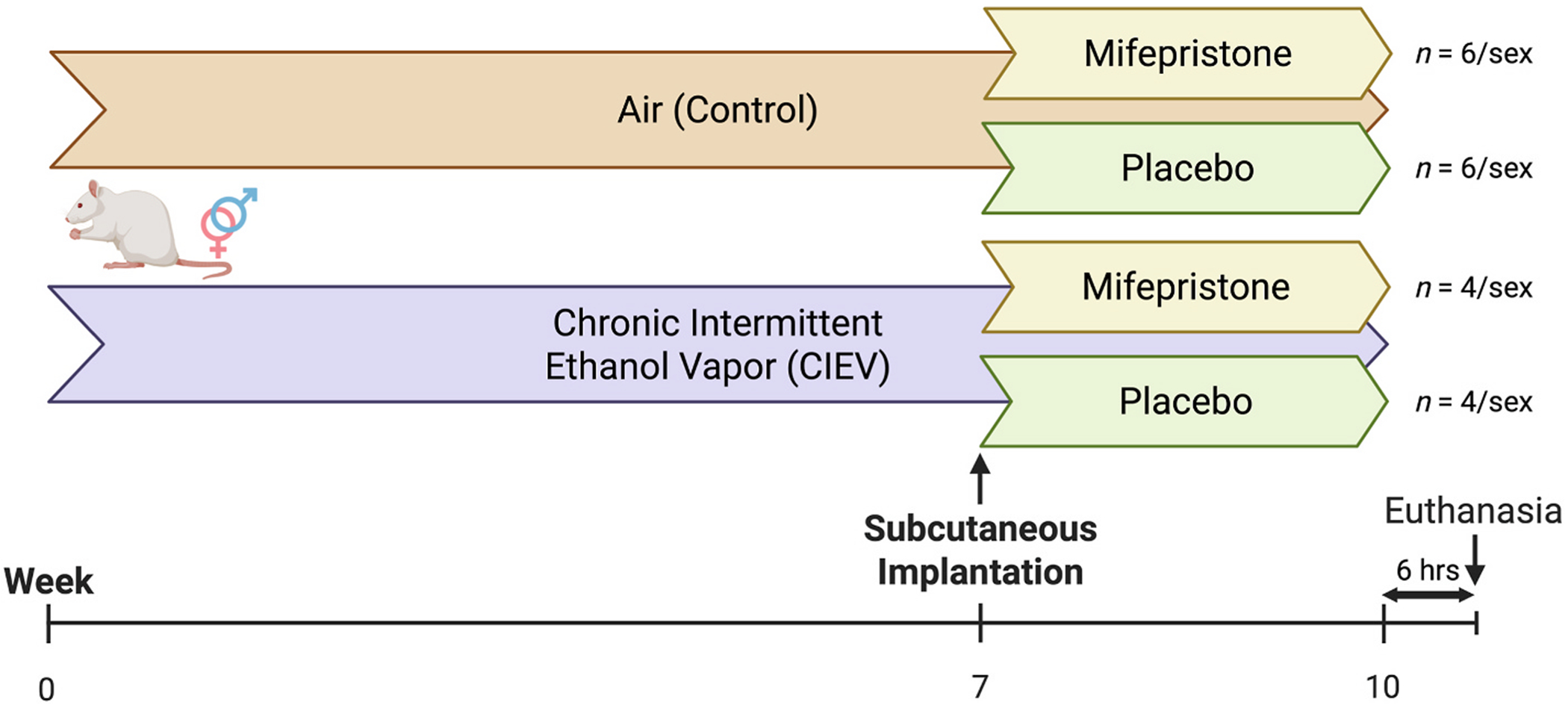
Experimental Timeline. Male and female Wistar rats were exposed to either air (control; *n* = 12/sex) or chronic intermittent ethanol vapor (CIEV; *n* = 8/sex) for 10 weeks. In week 7, half of each group received subcutaneous placebo pellets (*n* = 6/sex for controls; *n* = 4/sex for CIEV) while the other half received mifepristone pellets (150 mg, 21-day slow release; *n* = 6/control/sex; *n* = 4/CIEV/sex). At the end of week 10, the rats were euthanized during acute (6-h) CIEV withdrawal, and at an identical time in control groups. Created in BioRender.

The experimental timeline is shown in Fig. 1. Adult (8 weeks old upon arrival) female and male Wistar rats (N = 20/sex) were purchased from Charles River. Rats were pair-housed by sex and given *ad libitum* access to food (Purina Rat Chow, Ralston Purina) and water throughout the experiment. Rats were maintained on a reverse 12-h light/dark cycle (lights off at 8:00 a.m.), handled regularly, and allowed 1 week to acclimate to the colony room prior to the start of experimental procedures. All animal care, use, and procedures in this study were approved by the Institutional Animal Care and Use Committee of Louisiana State University Health Sciences Center at New Orleans (LSUHSC-New Orleans; IACUC #3839) and were in accordance with the National Institute of Health guidelines.

### 2.2. Chronic, intermittent ethanol vapor exposure

Rats were pair-housed in plexiglass vapor delivery chambers. Chronic, intermittent ethanol vapor (CIEV; “alcohol-dependent”) and control groups (air-exposed; “non-dependent”) were housed in separate rooms in individually ventilated cages. CIEV procedures in which animals undergo cycles of alcohol exposure and abstinence over several weeks were the same as previously described (Edwards et al., 2012; Nicholas W. Gilpin et al., 2008). The CIEV procedure includes daily cycles of 190 proof ethyl alcohol vapor exposure (14 h) and alcohol withdrawal (10 h), where blood alcohol levels (BALs) are reduced to near zero and physical and motivational withdrawal symptoms emerge. Tail blood samples were collected and analyzed one to two times per week to maintain BALs of 150–250 mg/dL, as previously described (Nicholas W. Gilpin et al., 2008). This procedure has been reliably used to produce both somatic and motivation-like symptoms of alcohol dependence, including escalation of alcohol consumption and emergence of negative affective behaviors and hyperalgesia in both male and female rodents (Bach et al., 2021; Brandner et al., 2023; Gilpin et al., 2008; Lopez et al., 2023; Miller et al., 2025; Rogers et al., 1979; Vendruscolo and Roberts, 2014). To assess BALs, tail blood (0.2 mL) was collected and centrifuged to extract the plasma. The plasma was injected into an oxygen-rated alcohol analyzer (Analox Instruments, London, UK) for BAL determination via an alcohol oxidation reaction as previously described (McGinn et al., 2016). Single-point calibrations were done for each set of samples with reagents provided by Analox Instruments (25–400 mg/dL). Non-dependent air control rats were not exposed to alcohol vapor. Group BAL averages over the last three weeks of exposure are as follows: Male-CIEV-Placebo (199.9 mg/dL), Male-CIEV-Mifepristone (202.6 mg/dL), Female-CIEV-Placebo (232.7 mg/dL), Female-CIEV-Mifepristone (225.7 mg/dL).

### 2.3. Mifepristone (RU-486) treatment

Half of the Wistar rats in each CIEV and air (control) group were subcutaneously implanted with mifepristone pellets (a GR/progesterone receptor antagonist; 150 mg; Innovative Research of America, Sarasota, FL) or placebo pellets for chronic release (21-day slow release). The mifepristone dose was chosen based on previous studies (Nephew et al., 2008; Schneider et al., 2003; Vendruscolo et al., 2012).

### 2.4. Brain collection and regional CeA tissue dissection

Brains from dependent and non-dependent Wistar rats were collected and snap-frozen with isopentane for proteomics analyses during an acute 6-h withdrawal from vapor exposure (or the identical time point in air-exposed animals). This time point was chosen based on previous studies showing an escalation of alcohol consumption and emergence of negative affective behaviors and heightened nociceptive sensitivity in rats at 6–8 h of withdrawal from ethanol vapor exposure (Gilpin et al., 2009; N. W. Gilpin et al., 2008; O’Dell et al., 2004; Rimondini et al., 2003; Rylkova et al., 2009; Vendruscolo et al., 2012), which were also observed in female rodents (Bach et al., 2021; Brandner et al., 2023; Cruise et al., 2025; Lopez et al., 2023; Miller et al., 2025). Thus, euthanasia at 6 h was optimal to capture CeA neuroadaptations associated with acute withdrawal in both sexes. The brains were sliced on a cryostat, and bilateral punches (1.0 mm thickness from −1.7 mm to −2.7 mm bregma, 16-gauge diameter) were collected from the central amygdala (CeA). CeA tissue punches were collected from individual animals, and the samples were not pooled. The number of biological replicates per group was as follows: n = 6/sex for Air-Placebo and Air-Mifepristone and n = 4/sex for CIEV-Placebo and CIEV-Mifepristone.

### 2.5. Quantitative discovery-based proteomics using tandem mass tags and liquid chromatography – mass spectrometry

Samples were prepared for quantitative discovery-based quantitative proteomic analysis by adding 1 % SDS and sonication until they were completely homogeneous. The protein concentration was determined using a BCA Protein Assay Kit (Thermo Fisher Scientific, Waltham, MA, USA). Based on the protein concentration, 100 μg of each sample was prepared for trypsin digestion by reducing the cysteines with tris(2-carboxyethyl)phosphine, followed by alkylation with iodoacetamide. After chloroform–methanol precipitation, each protein pellet was digested with trypsin overnight at 37 °C. The digested product was labeled using a TMT11-Plex Isobaric Label Reagent Set (Thermo Fisher Scientific) according to the manufacturer’s protocol and stored at −80 °C until further use. An equal amount of each tandem mass tag (TMT)-labeled sample was pooled together in a single tube and Sep-Pak purified (Waters Chromatography Ireland, Ltd., Dublin, Ireland) under acidic reversed-phase conditions. After labeling, a C18 cleanup step was performed to remove any unbound TMT labels, followed by offline fractionation prior to LC-MS injection. The resulting fractions were pooled to separate peptides by polarity across injections, thereby improving peptide identifications and reducing the masking of low-abundance peaks by highly abundant peaks. After drying to completion, an offline fractionation step was employed to reduce the complexity of the sample. The sample was brought up in 10-mM ammonium hydroxide, pH 10. This mixture was subjected to basic pH reversed-phase chromatography using a Dionex UltiMate 3000 (Thermo Fisher Scientific). Briefly, the fractions were monitored by ultraviolet (UV) at 215 nm for an injection of 100 μL at 0.1 mL/min, with a gradient developed from 10 mM ammonium hydroxide (pH 10) to 100 % acetonitrile (ACN) over 90 min. A total of 48 fractions (200 μL each) were collected in a 96- well microplate and recombined in a checker-board fashion to create 12 “super fractions” (original fractions 1, 13, 25, and 37 became new super fraction 1, etc.; original fractions 2, 14, 26, and 38 became new super fraction 2, etc.). The 12 super fractions were then run on a Thermo Fisher Scientific UltiMate 3000 nano-flow system coupled to a Thermo Fisher Scientific Fusion Orbitrap mass spectrometer.

Each fraction was subjected to a 95-min chromatographic method employing a gradient from 1 % to 32 % ACN in 0.1 % formic acid (ACN/FA) for 70 min, a gradient to 50 % ACN/FA for an additional 10 min, a step to 99 % ACN/FA for 3 min, and a 12-min re-equilibration into 1 % ACN/FA. Chromatography was carried out in a “trap-and-load” format. The trap column was an Acclaim C18 PepMap100, 5 μm, 100 Å, and the column was a PepMap RSLC C18 2 μm, 100 Å, 75 μm × 25 cm (Thermo Fisher, ES902). The entire run was conducted at a 0.3 μl/min flow rate, and the sample was ionized through a Thermo Easyspray ion source.

TMT data acquisition utilized an MS3 approach for data collection. Survey scans (MS1) were performed in the Orbitrap with a resolution of 120,000 with a scan range of 375 m/z to 1600 m/z. Data-dependent MS2 scans were performed in the linear ion trap using a collision-induced dissociation of 25 %. Reporter ions were fragmented using high-energy collision dissociation (HCD) of 55 % and detected in the Orbitrap with a resolution of 50,000. The automatic gain control (AGC) target was set to the standard level. This was repeated for a total of three technical replicates.

### 2.6. Bioinformatic and principal component analyses

Male and female TMT data analyses were performed using Proteome Discoverer 2.4 with SEQUEST HT scoring separately. The protein FASTA database was for *Rattus norvegicus* (SwissProt TaxID = 10116) (version 2017-10-25). Static modifications included TMT reagents on lysine and N-terminus (+229.163 Da) and carbamidomethyl on cysteines (+57.021 Da). Dynamic modification included oxidation of methionine (+15.9949 Da) and phosphorylation of Serine, Threonine, and Tyrosine (+79.966 Da). Only high-scoring peptides were considered, utilizing a false discovery rate of 1 %.

Abundance ratios were calculated by pairwise comparisons of experimental/control groups. Pairwise comparisons are shown in Table 1. Results from pairwise comparisons of CIEV-Placebo versus Air-Placebo groups are referred to as CIEV-mediated effects, and a complete list of significantly differentially expressed proteins is listed in Supplemental Table 1 for male rats and Supplemental Table 6 for female rats. Results from pairwise comparisons of Air-Mifepristone versus Air-Placebo groups are referred to as Mifepristone-mediated effects, and a complete list of significantly differentially expressed proteins is listed in Supplemental Table 2 for male rats and Supplemental Table 7 for female rats. Results from pairwise comparisons of CIEV-Mifepristone versus Air-Mifepristone groups are referred to as CIEV-mediated effects in the context of mifepristone, and a complete list of significantly differentially expressed proteins is listed in Supplemental Table 3 for male rats and Supplemental Table 8 for female rats. Results from pairwise comparisons of CIEV-Mifepristone versus CIEV-Placebo groups are referred to as Mifepristone-mediated effects in the context of CIEV, and a complete list of significantly differentially expressed proteins is listed in Supplemental Table 4 for male rats and Supplemental Table 9 for female rats. Statistical testing was performed using an unpaired two-tailed *t*-test. *P* values were adjusted using the False Discovery Rate (FDR) method separately for each comparison group. Proteins with at least a 1.25/1-fold change increase or 1/1.25-fold change decrease with an adjusted *P* value (FDR) of 0.05 were considered significant changes.

**Fig. 2.**
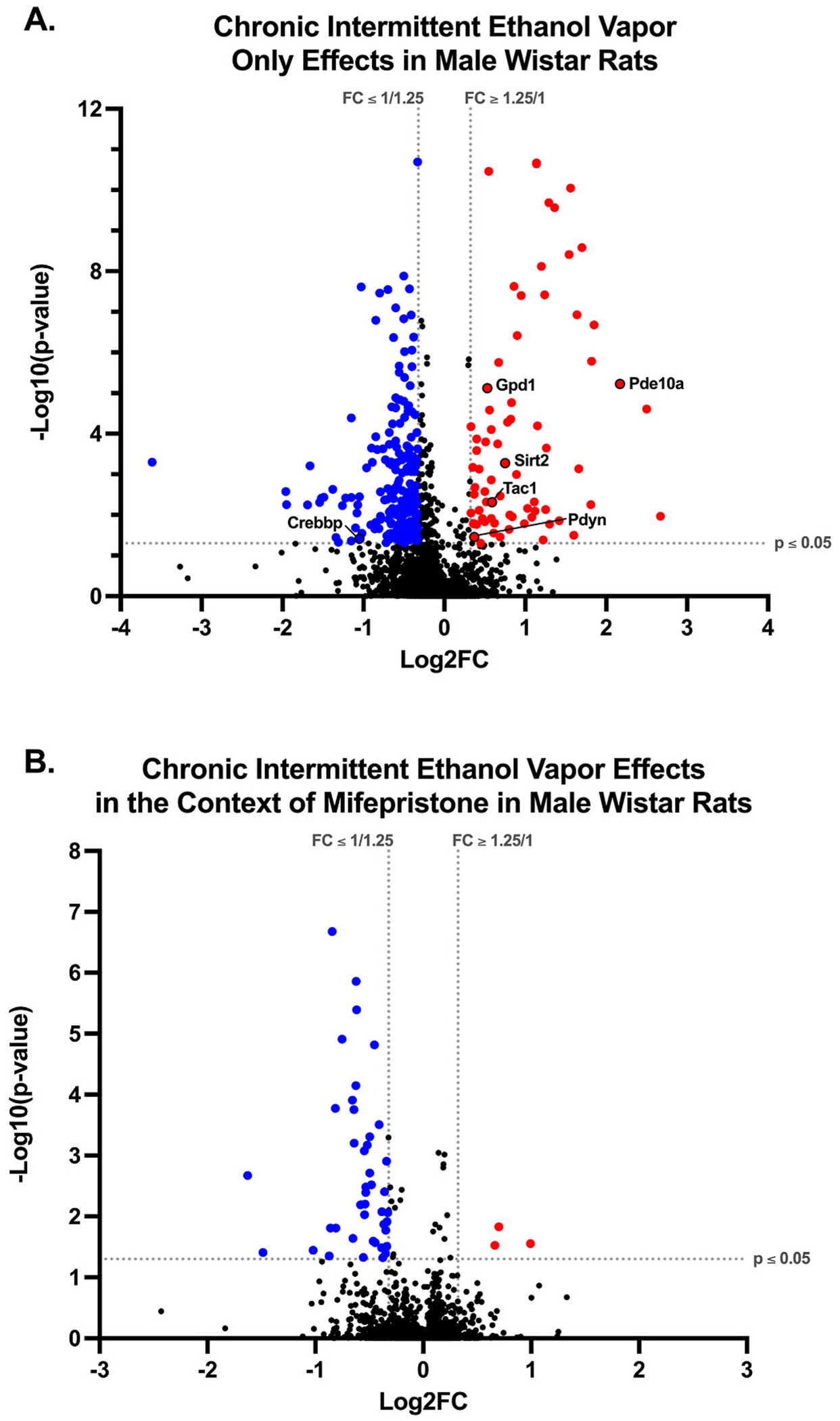
Effects of Chronic Intermittent Ethanol Vapor in Males. Graphical representations of male Wistar rat central amygdala (CeA) proteomics data based on Chronic Intermittent Ethanol Vapor (CIEV) alone (*A*; CIEV-Placebo vs. Air-Placebo) and CIEV in the context of mifepristone (*B*; CIEV-Mifepristone vs. Air-Mifepristone). Volcano plots of proteomics data based on CIEV effects are shown as *P* values vs. fold change (FC). Colored points in the volcano plots represent data points with *P* ≤ 0.05 and an FC ≥ 1.25/1 (red) or FC ≤ 1/1.25 (blue). (For interpretation of the references to colour in this figure legend, the reader is referred to the Web version of this article.)

**Fig. 3.**
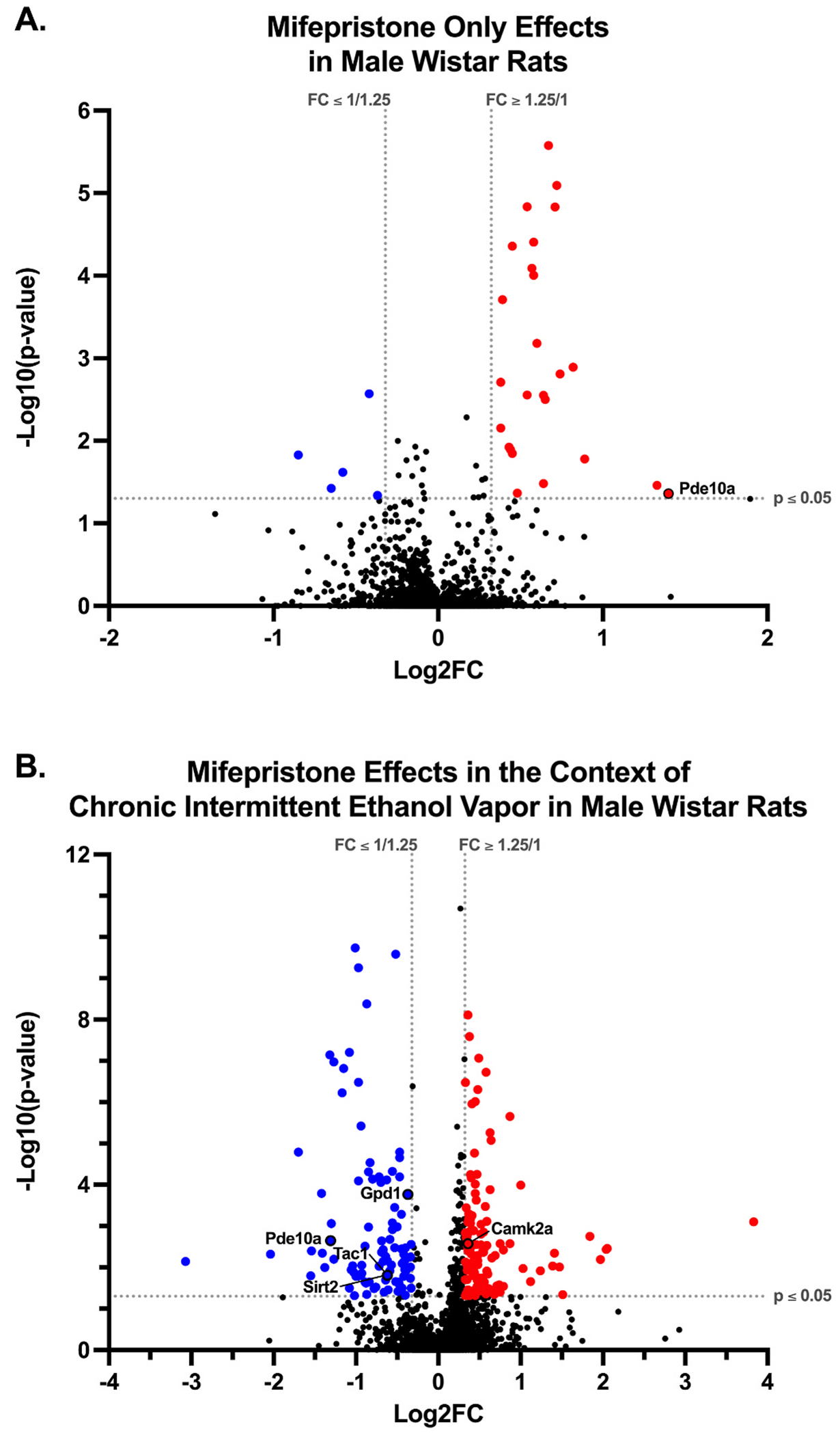
Effects of Mifepristone in Males. Graphical representations of male Wistar rat central amygdala (CeA) proteomics data based on mifepristone treatment alone (*A*; Air-Mifepristone vs. Air-Placebo) and mifepristone treatment in the context of CIEV (*B*; CIEV-Mifepristone vs. CIEV-Placebo). Volcano plots of proteomics data based on mifepristone effects are shown as *P* values vs. fold change (FC). Colored points in the volcano plots represent data points with a *P* ≤ 0.05 and an FC ≥ 1.25/1 (red) or FC ≤ 1/1.25 (blue). (For interpretation of the references to colour in this figure legend, the reader is referred to the Web version of this article.)

**Fig. 4.**
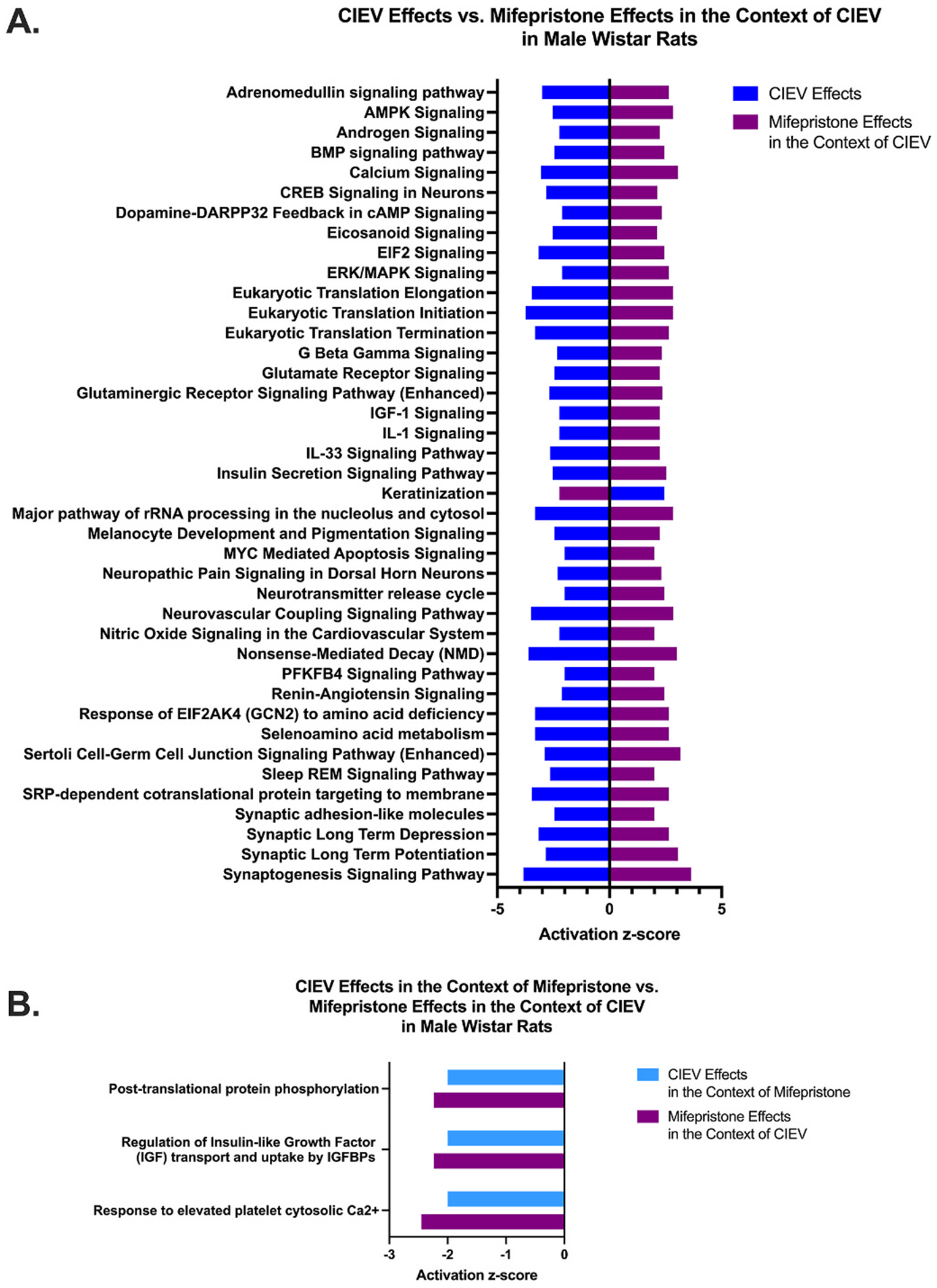
Ingenuity Canonical Pathways Altered in Males. Overlapping ingenuity canonical pathways of greatest predicted activation (positive value; blue) or inhibition (negative value; red) associated with protein changes in response to CIEV vs. CIEV in the context of mifepristone (*A*) and CIEV in the context of mifepristone vs. mifepristone in the context of CIEV (B) with calculated activation z-scores in the CeA of adult male Wistar rats. (For interpretation of the references to colour in this figure legend, the reader is referred to the Web version of this article.)

**Fig. 5.**
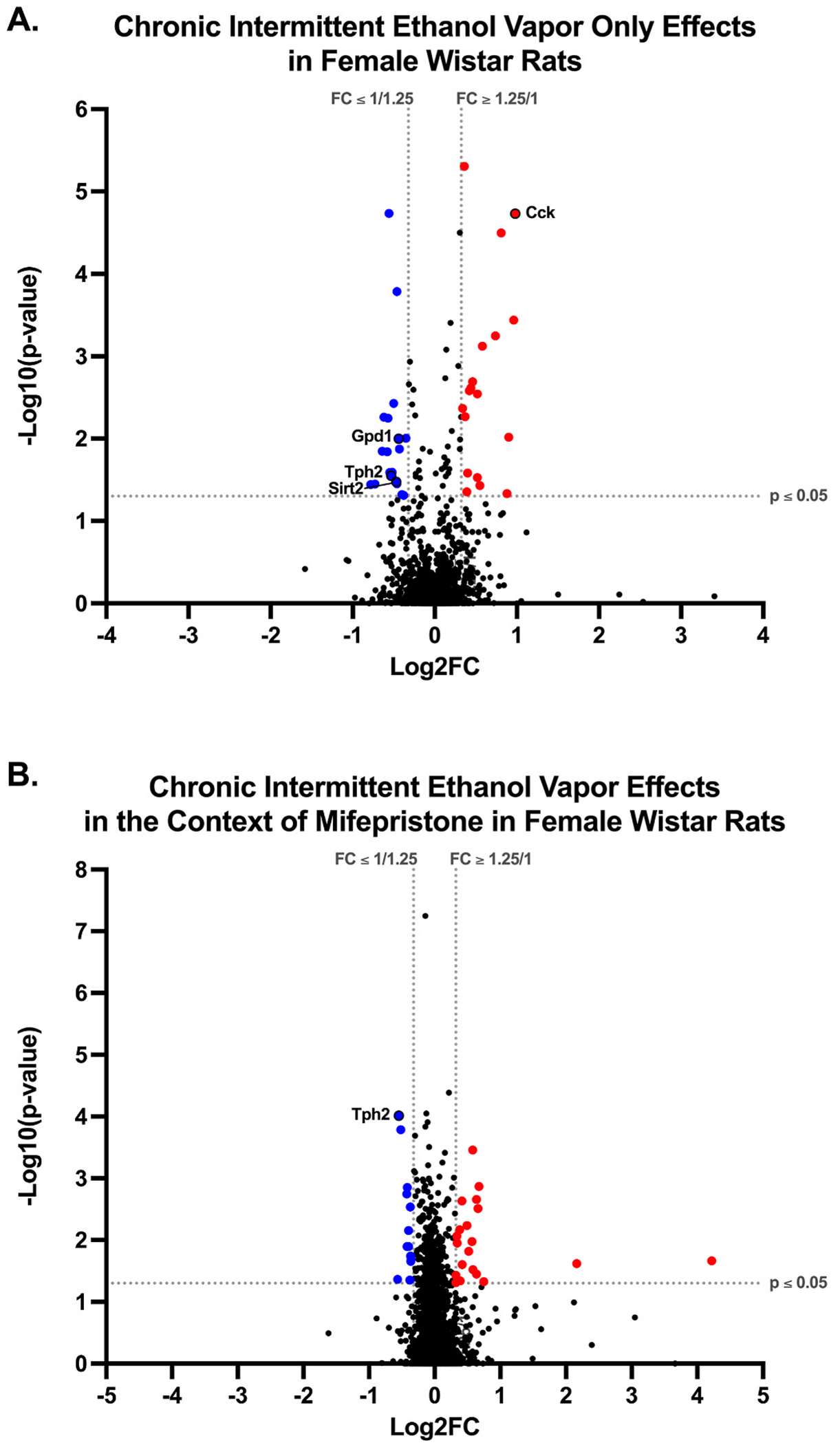
Effects of Chronic Intermittent Ethanol Vapor in Females. Graphical representations of female Wistar rat central amygdala (CeA) proteomics data based on Chronic Intermittent Ethanol Vapor (CIEV) alone (*A*; CIEV-Placebo vs. Air-Placebo) and CIEV in the context of mifepristone (*B*; CIEV-Mifepristone vs. Air-Mifepristone). Volcano plots of proteomics data based on CIEV effects are shown as *P* values vs. fold change (FC). Colored points in the volcano plots represent data points with *P* ≤ 0.05 and an FC ≥ 1.25/1 (red) or FC ≤ 1/1.25 (blue). (For interpretation of the references to colour in this figure legend, the reader is referred to the Web version of this article.)

**Fig. 6.**
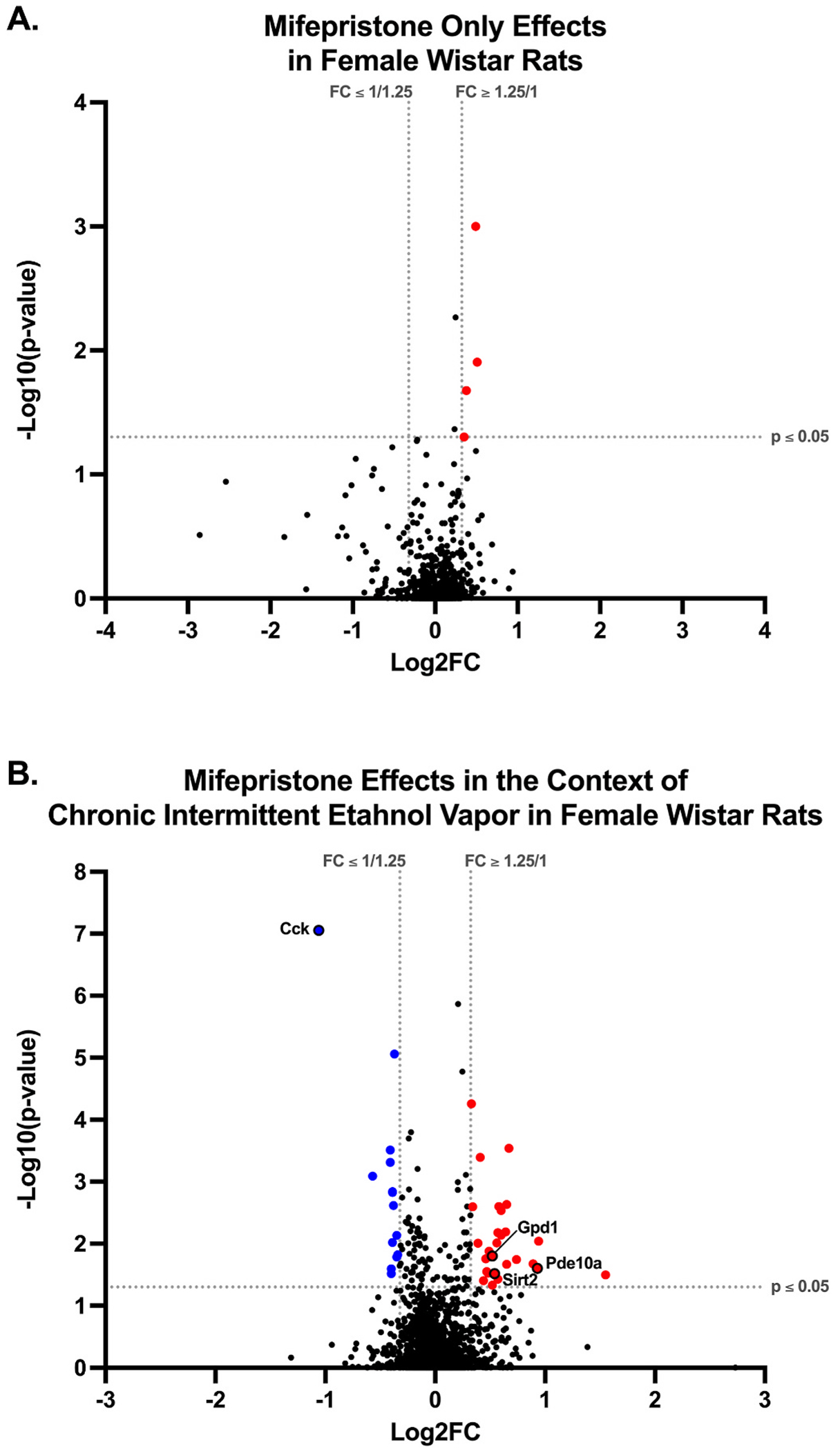
Effects of Mifepristone in Females. Graphical representation of female Wistar rat central amygdala (CeA) proteomics data based on mifepristone treatment alone (*A*; Air-Mifepristone vs. Air-Placebo) and mifepristone treatment in the context of CIEV (*B*; CIEV-Mifepristone vs. CIEV-Placebo). Volcano plots of proteomics data based on mifepristone effects are shown as *P* values vs. fold change (FC). Colored points in the volcano plots represent data points with *P* ≤ 0.05 and an FC ≥ 1.25/1 (red) or FC ≤ 1/1.25 (blue). (For interpretation of the references to colour in this figure legend, the reader is referred to the Web version of this article.)

**Fig. 7.**
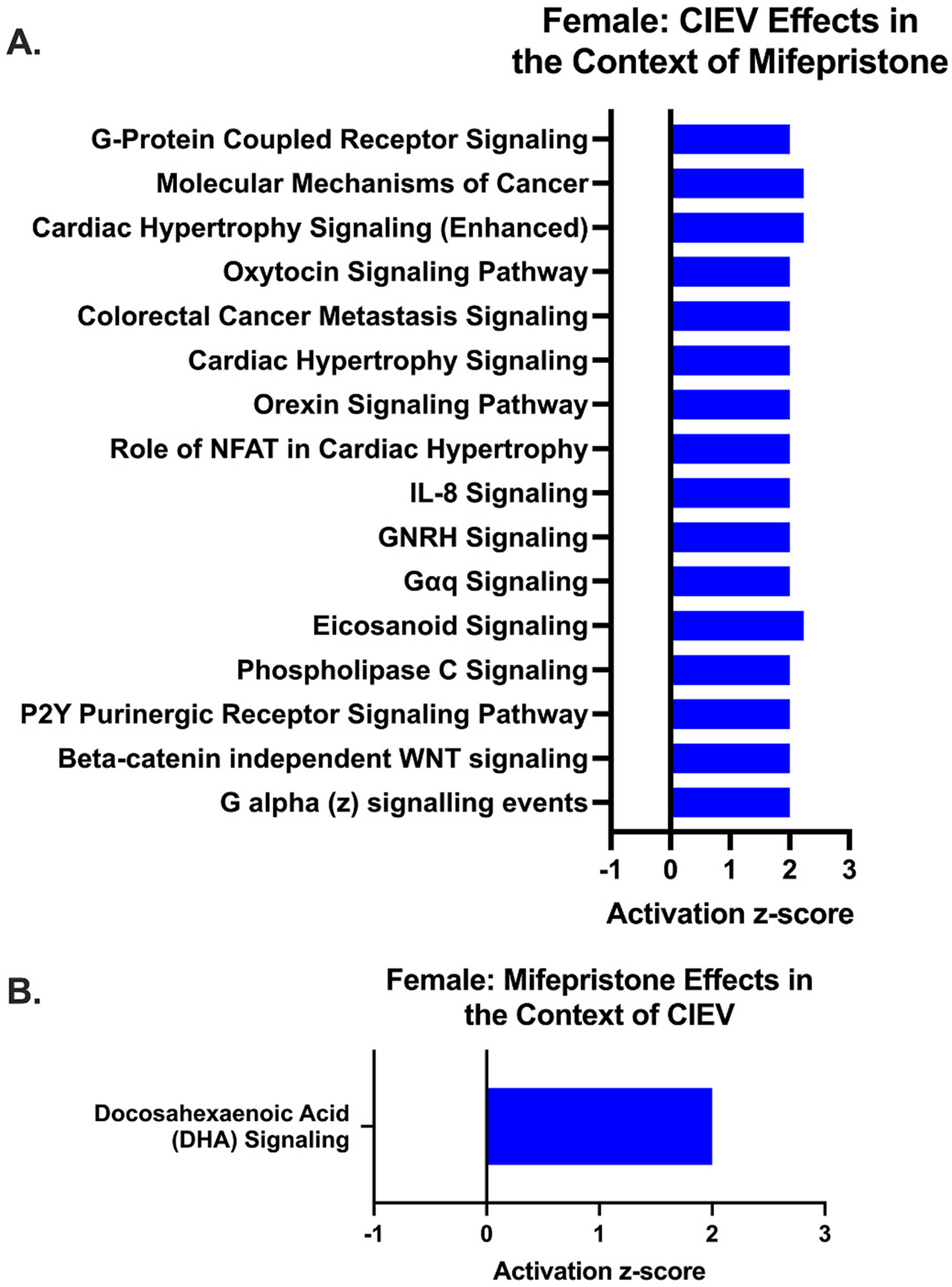
Ingenuity Canonical Pathways Altered in Females. Ingenuity canonical pathways of greatest predicted activation (positive value; blue) or inhibition (negative value; red) associated with protein changes in response to CIEV in the context of mifepristone (*A*) and mifepristone treatment in the context of CIEV (*B*) with calculated activation z-scores in the CeA of adult female Wistar rats. Only 1 canonical pathway was significantly altered by mifepristone in the context of CIEV. No significant changes to canonical pathways were observed in response to CIEV or mifepristone alone in adult female Wistar rats. (For interpretation of the references to colour in this figure legend, the reader is referred to the Web version of this article.)

**Fig. 8.**
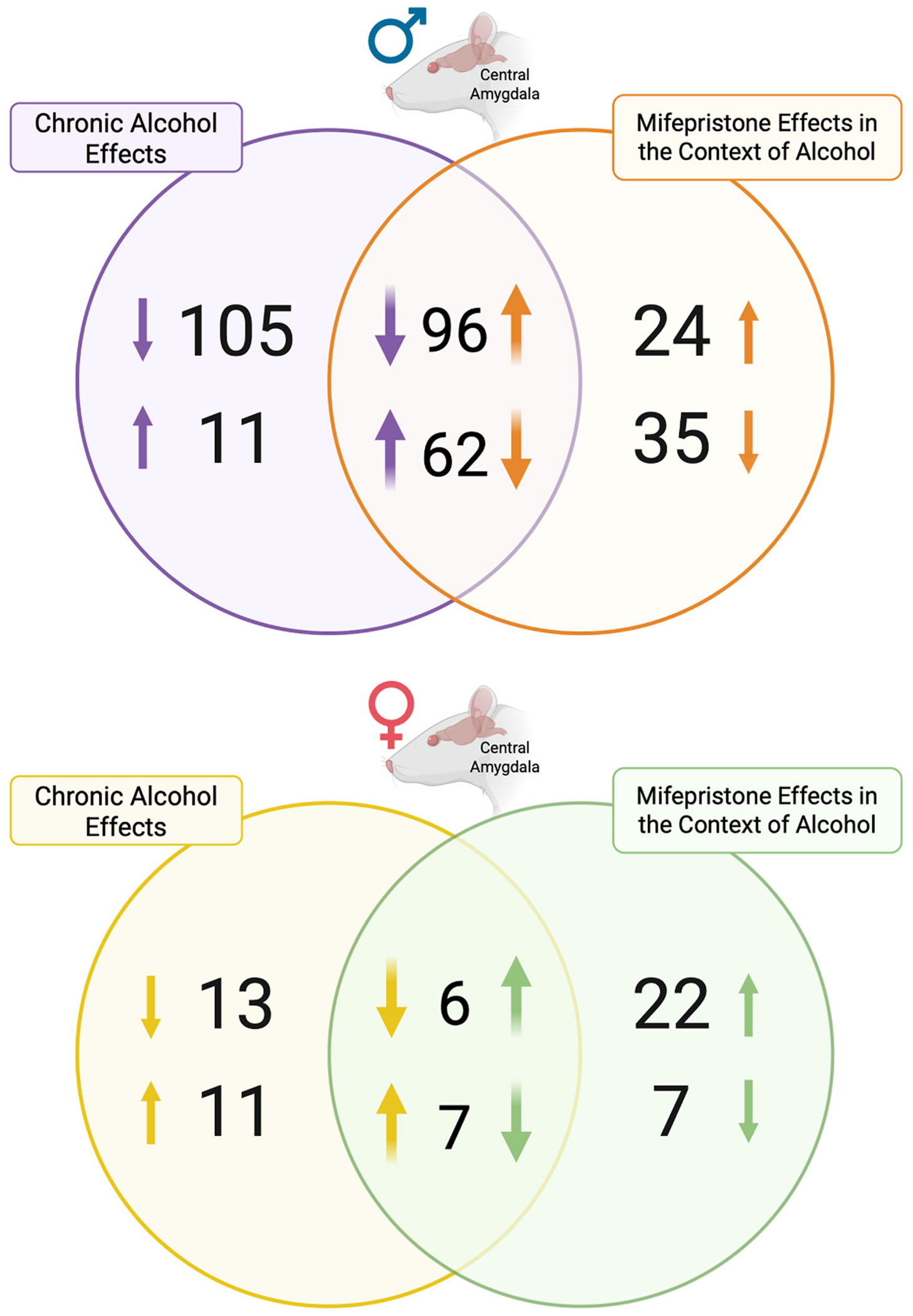
Numerical Summary of Protein Changes in Male and Female Wistar Rat Central Amygdala. CIEV significantly downregulated 201 proteins and upregulated 73 proteins in male Wistar rats. Mifepristone in the context of CIEV downregulated 97 proteins, 62 of which were upregulated by CIEV, and upregulated 120 proteins, 96 of which were downregulated by CIEV, in male Wistar rats. CIEV significantly downregulated 19 proteins and upregulated 18 proteins in female Wistar rats. Mifepristone in the context of CIEV downregulated 14 proteins, 7 of which were upregulated by CIEV, while upregulating 28 proteins, 6 of which were downregulated by CIEV, in female Wistar rats. Created in BioRender.

Bioinformatic analyses were performed using Qiagen Ingenuity Pathway Analysis (IPA) software (QIAGEN Inc., https://www.qiagenbioinformatics.com/products/ingenuity-pathway-analysis). Proteins with at least a 1.25-fold change (up or down) and an adjusted *P* value (FDR) of 0.05 were considered in the core analysis to identify alterations in canonical pathways. In an expression analysis, IPA calculated *z*-scores, or activation predictions, for a functional annotation. Only those pathways with |z-score| ≥ 2 were considered significant. A complete list of significantly altered canonical pathways is listed in Supplemental Table 5 for male rats and Supplemental Table 10 for female rats. Unlike the *P* value, the *z*-score considers the direction of protein expression. A negative *z*-score indicates a functional activity or pathway that is inhibited, and a positive *z*-score indicates functional activity or pathway activation. Principal component analysis (PCA) was performed on proteomic data of over 3000 proteins using the prcomp function within the R package stats (version 3.6.2; Figs. S1 and S2). Figures were created in BioRender.com (Toronto, Canada; Figs. 1 and 8) or with GraphPad Prism 10 for macOS Version 10.5.0 (San Diego, CA; Figs. 2–7).

## 3. Results

### 3.1. CIEV and mifepristone differentially alter CeA proteomic profile of male Wistar rats

A total of 3050 proteins were identified and quantified by proteomic profiling of male Wistar rat CeA samples. Volcano plots of differentially regulated proteins by CIEV alone (Fig. 2A) and CIEV in the context of mifepristone (Fig. 2B) are shown in Fig. 2, and the proteins of interest are labeled. The red circles depict data with significant P values (≤ 0.05) and an absolute fold change (FC) value of equal to or greater than 1.25/1, and the blue circles for those with significant P values (≤ 0.05) an absolute FC value of equal to or less than 1/1.25 when comparing CIEV-Placebo versus Air-Placebo (Fig. 2A) or CIEV-Mifepristone versus Air-Mifepristone (Fig. 2B).

CIEV alone (CIEV-Placebo vs. Air-Placebo) significantly down-regulated 201 proteins and upregulated 73 proteins, as shown in Supplemental Table 1. CIEV in the context of mifepristone (CIEV-Mifepristone vs. Air-Mifepristone) significantly downregulated 42 proteins and upregulated 3 proteins (Supplemental Table 2). CIEV in the two comparisons (CIEV-Placebo vs. Air-Placebo and CIEV-Mifepristone vs. Air-Mifepristone) upregulated 1 common protein (Protein os-9) but did not downregulate any common proteins. CIEV in the two comparisons regulated 19 other common proteins, although the direction of protein expression changes occurred in opposite directions.

Volcano plots of differentially regulated proteins by mifepristone in male Wistar rats are shown in Fig. 3, and the proteins of interest are labeled. The red circles depict data with significant P values (≤0.05) and an absolute fold change (FC) value of equal to or greater than 1.25/1, and the blue circles for those with significant P values (≤0.05) an absolute FC value of equal to or less than 1/1.25 when comparing Air-Mifepristone versus Air-Placebo (Fig. 3A) or CIEV-Mifepristone versus CIEV-Placebo (Fig. 3B).

Mifepristone alone significantly downregulated 5 proteins and upregulated 25 proteins, as shown in Supplemental Table 3. Mifepristone in the context of CIEV (CIEV-Mifepristone vs. CIEV-Placebo) significantly downregulated 97 proteins and upregulated 120 proteins (Supplemental Table 4). Mifepristone treatment in the two comparisons regulated 23 common proteins, although in opposite directions (i.e., upregulated by mifepristone alone but downregulated by mifepristone in the context of CIEV), suggesting that mifepristone differentially regulates CeA proteins in the context of alcohol dependence. For example, mifepristone upregulated cAMP and cAMP-inhibited cGMP 3′,5′-cyclic phosphodiesterase 10A (Pde10a) in control animals while down-regulating it in alcohol-dependent male Wistar rats.

Interestingly, 158 of 274 proteins altered by CIEV were “normalized” by mifepristone treatment, where 96 of these 158 proteins that were significantly downregulated by CIEV were consequently significantly upregulated by mifepristone treatment in the context of CIEV exposure, and 62 of those 158 proteins that were upregulated by CIEV were consequently downregulated by mifepristone treatment in the context of CIEV exposure. In this study, we use the terms “normalization” and “reversal” to describe cases where protein expression altered by CIEV was significantly shifted back toward levels observed in Air controls following mifepristone treatment. These refer to instances where proteins altered in one direction by CIEV were subsequently shifted in the opposite direction by mifepristone. Importantly, these interpretations are supported by IPA z-scores, which consider both magnitude and direction of protein expression and confirm that many of these apparent reversals correspond to statistically meaningful corrections at the pathway level as well.

### 3.2. Principal component analysis of proteomics data of male Wistar rats

To better understand how CIEV and mifepristone affect the CeA proteome, we utilized PCA to compare the proteomic profiles from all four treatment groups. PCA analysis revealed two principal components (PCs) that best accounted for variability in our data set (Supplemental Fig. 1). In male Wistar rats, PC1 accounts for 72.1 % of variability, whereas PC2 accounts for 17.3 % of variability. In males, the Euclidean distance between groups was the greatest between the Air-Placebo and CIEV-Placebo groups, while the Air-Placebo and Air-Mifepristone had the shortest distance. These observations suggest that the CeA proteome profile is most substantially impacted by CIEV administration, and that mifepristone administration appears to diminish the effects of CIEV on the CeA proteome profile.

### 3.3. Ingenuity canonical pathways affected by CIEV and mifepristone in male Wistar rats

To understand functional mechanisms associated with CIEV-mediated differentially expressed proteins, the proteomic data set was submitted to IPA core analysis (Krämer et al., 2014). The top-enriched categories of canonical pathways with a *P* value less than 0.05, the −log(*p*-value), ratio, activation z-score, and representative differentially expressed proteins in each canonical pathway are listed in Supplemental Table 5. There were no significant changes to canonical pathways in response to mifepristone treatment alone in adult male Wistar rats.

70 canonical pathways were affected by CIEV alone; 5 pathways were affected by CIEV in the context of mifepristone; 43 canonical pathways were affected by mifepristone treatment in the context of CIEV (Supplemental Table 5).

### 3.4. Directionality of effects on pathways affected by CIEV vs. mifepristone in the context of CIEV and CIEV in the context of mifepristone vs. mifepristone in the context of CIEV in male Wistar rats

IPA core analysis provides *z*-score values indicating predicted pathway activation (positive values) or inhibition (negative values). Fig. 4 illustrates canonical pathways affected in multiple comparisons, accompanied by a calculated *z*-score for each comparison.

Both CIEV and mifepristone altered a total of 40 canonical pathways in the context of CIEV (Fig. 4A). 39 of these canonical pathways were downregulated by CIEV but upregulated by mifepristone treatment in the context of CIEV, and 1 canonical pathway was upregulated by CIEV but downregulated by mifepristone treatment in the context of CIEV (Fig. 4A), suggesting that mifepristone corrects canonical pathways affected by CIEV exposure.

A total of 3 canonical pathways were altered by both CIEV in the context of mifepristone and mifepristone in the context of CIEV (Fig. 4B). All of these pathways were downregulated by both CIEV in the context of mifepristone and mifepristone in the context of CIEV.

### 3.5. CIEV and mifepristone differentially alter CeA proteomic profile of female Wistar rats

A total of 2631 proteins were identified and quantified by proteomic profiling of female Wistar rat CeA samples. Volcano plots of differentially regulated proteins by CIEV are shown in Fig. 5, and the proteins of interest are labeled. The red circles depict data with significant P values (≤ 0.05) and an absolute fold change (FC) value of equal to or greater than 1.25/1, and the blue circles for those with significant P values (≤0.05) an absolute FC value of equal to or less than 1/1.25 when comparing CIEV-Placebo versus Air-Placebo (Fig. 5A) or CIEV-Mifepristone versus Air-Mifepristone (Fig. 5B).

CIEV alone significantly downregulated 19 proteins and upregulated 18 proteins, as shown in Supplemental Table 6. CIEV in the context of mifepristone (CIEV-Mifepristone vs. Air-Mifepristone) significantly downregulated 13 proteins and upregulated 20 proteins (Supplemental Table 7). CIEV in the two comparisons downregulated 3 common proteins (Tryptophan 5-hydroxylase 2, Galectin-1, and ADP-ribosyl cyclase/cyclic ADP-ribose hydrolase 1) but did not upregulate any common proteins.

Volcano plots of differentially regulated proteins by mifepristone in male Wistar rats are shown in Fig. 6, and the proteins of interest are labeled. The red circles depict data with significant P values (≤0.05) and an absolute fold change (FC) value of equal to or greater than 1.25/1, and the blue circles for those with significant P values (≤0.05) an absolute FC value of equal to or less than 1/1.25 when comparing Air-Mifepristone versus Air-Placebo (Fig. 6A) or CIEV-Mifepristone versus CIEV-Placebo (Fig. 6B). Mifepristone alone did not significantly down-regulate any proteins in the female rat CeA (Fig. 6A).

Mifepristone alone significantly upregulated 4 proteins, as shown in Supplemental Table 8. Mifepristone in the context of CIEV (CIEV-Mifepristone vs. CIEV-Placebo) significantly downregulated 14 proteins and upregulated 28 proteins (Supplemental Table 9). Mifepristone treatment in the two comparisons did not regulate any common proteins.

Interestingly, 13 of 37 proteins altered by CIEV were “normalized” by mifepristone treatment, where 6 of these 13 proteins that were downregulated by CIEV were consequently upregulated by mifepristone treatment in the context of CIEV exposure, and 7 of those 13 proteins that were upregulated by CIEV were consequently downregulated by mifepristone treatment in the context of CIEV exposure.

### 3.6. Principal component analysis of proteomics data of female Wistar rats

PCA analysis was used to compare the CeA proteomic profiles of female Wistar rats (Supplemental Fig. 2). In females, PC1 accounted for 56.2 % of the variance, whereas PC2 accounted for 36.6 % of the variance. Similar to male rats, the smallest difference in Euclidean distance was between the Air-Placebo and Air-Mifepristone groups. The largest Euclidean distance across groups was between the Air-Mifepristone and CIEV-Placebo groups. PCA analysis indicates that mifepristone treatment has a greater impact on the CeA proteome of CIEV rats compared to Air (control) rats in females.

### 3.7. Ingenuity canonical pathways affected by CIEV and mifepristone in female Wistar rats

The top-enriched categories of canonical pathways with a *P* value less than 0.05, the -log(*p*-value), ratio, activation z-score, and representative differentially expressed proteins in each canonical pathway are listed in Supplemental Table 10.

16 pathways were affected by CIEV in the context of mifepristone (Fig. 7A), and 1 canonical pathway was affected by mifepristone treatment in the context of CIEV (Fig. 7B). A comprehensive list of canonical pathways altered in each comparison is depicted in Supplemental Table 10. There were no significant changes to canonical pathways in response to CIEV alone and mifepristone alone in adult female Wistar rats.

### 3.8. Directionality of effects on pathways in female Wistar rats

IPA core analysis provides *z*-score values indicating predicted pathway activation (positive values) or inhibition (negative values). Fig. 7 illustrates the canonical pathways, accompanied by a calculated *z*-score for each comparison.

CIEV in the context of mifepristone activated a total of 16 canonical pathways, and 1 canonical pathway was activated by mifepristone in the context of CIEV. However, there was no overlap in the canonical pathways altered in the two comparisons.

## 4. Discussion and conclusion

We examined the effects of alcohol dependence (CIEV exposure) and mifepristone treatment on the central amygdala (CeA) proteome in both male and female adult Wistar rats. Our quantitative, discovery-based proteomic approach identified potentially significant sex-specific differences in CeA protein expression profiles and changes due to CIEV, mifepristone, and their combination (Fig. 8).

About 9 % of the proteins detected within the CeA were significantly regulated by chronic alcohol exposure, with nearly 60 % of these proteins corrected by mifepristone treatment in adult male Wistar rats. In contrast, only 1 % of the proteins were significantly regulated by chronic alcohol exposure in adult female Wistar rats, with approximately 35 % of those normalized by mifepristone. Although no direct comparisons were made between the male and female rats, the data collectively suggest that chronic ethanol vapor exposure and mifepristone treatment modulate CeA protein expression more robustly in males than in females. This may reflect an underlying sex-dependent sensitivity of the CeA to alcohol and stress hormone (cortisol) regulation.

Individuals with alcohol use disorder (AUD) often exhibit elevated basal cortisol levels (Wand and Dobs, 1991), while rodent models similarly show elevated basal corticosterone levels during intoxication and withdrawal (Rivier et al., 1983; Tabakoff et al., 1978). Alcohol-induced glucocorticoid release facilitates a pathological loop between stress and reinforcement circuitry. The CeA is hypothesized to be a critical hub for the establishment of AUD as well as numerous AUD-related negative affective co-morbidities. As one example, alcohol-induced hyperalgesia is a common symptom of alcohol dependence (Egli et al., 2012; Jochum et al., 2010; Zale et al., 2015), and mifepristone has been shown to be effective in alleviating or even preventing hyperalgesia symptoms in alcohol-dependent male rats (Dina et al., 2008). Importantly, mifepristone has also been shown to reduce alcohol drinking in high-stress, limited-access conditions (Vendruscolo et al., 2015; Koenig and Olive, 2004) as well as prevent the escalation of drinking in the context of dependence (Vendruscolo et al., 2012). These dramatic effects suggest an efficacy with both acute and chronic treatments, with the latter presumably via mifepristone’s ability to gate a wide range of transcriptional events and downstream targets involved in stress-related neurobiology. For example, corticotropin-releasing factor (CRF) is a key neuropeptide regulated by the GR that is produced in the hypothalamus and other brain regions, activating the HPA stress axis. The CeA is another major source of CRF, and due to its significant roles in pain, emotion, and negative reinforcement, the hyperactivation of the GR-linked CRF systems in the CeA is hypothesized to increase hyperalgesia, negative affect, and alcohol drinking during withdrawal in the context of alcohol dependence (Roberto et al., 2021). The current study set out to determine additional mifepristone-regulated systems that are altered in the context of alcohol dependence. Selected candidate systems are discussed below.

### 4.1. Adaptations in CaMKII/CREB signaling

In adult male Wistar rats, chronic intermittent ethanol vapor (CIEV) led to a suppression of cAMP response element-binding protein (CREB) signaling in the CeA (activation z-score = −2.828; Supplemental Table 5), a pathway known to regulate emotional response, synaptic plasticity, and stress resilience (Wand, 2005; Tronson et al., 2012; Harada et al., 2024; Sakamoto et al., 2011; Manners et al., 2019). Reductions in CeA CREB transcriptional activity in the context of chronic alcohol exposure and inherent propensity for alcohol drinking have been elegantly described (Pandey et al., 1999, 2005), and evidence even suggests that alterations in CREB activity produced by adolescent alcohol exposure may persist into adulthood (Zhang et al., 2018). Importantly, mifepristone treatment in the context of CIEV restored CREB signaling (activation z-score = 2.138; Fig. 4A; Supplemental Table 5), including the upregulation of calcium/calmodulin-dependent protein kinase II alpha (CaMKIIa; Log2FC = 0.36; Supplemental Table 4), a critical kinase that activates CREB to promote transcription of genes involved in neuronal survival, memory, and synaptic efficacy (Bok et al., 2007; Yang et al., 2025; Zalcman et al., 2018; Moriguchi et al., 2011). This restoration of CREB signaling may underlie the observed behavioral effects of mifepristone and represents another potential transcriptional target through which stress signaling intersects with reinforcement and mood regulation.

### 4.2. PDE10A

PDE10A (cAMP and cAMP-inhibited cGMP 3′,5′-cyclic phosphodiesterase 10A) is a dual specificity cAMP and cGMP hydrolyzing enzyme that was initially found to be increased in the amygdala following stress exposure in relation to alcohol self-administration levels (Logrip and Zorrilla, 2012). Subsequent work discovered widespread functional increases in PDE10A gene expression across multiple brain regions in the context of stress and alcohol exposure (Logrip et al., 2014; Logrip and Zorrilla, 2014), with brain PDE10A expression influenced by stress history and sex. Similarly, the current study found increased levels of CeA PDE10A in CIEV-exposed male rats (Log2FC = 2.17; Supplemental Table 1), which were normalized by mifepristone treatment (Log2FC = −1.31; Supplemental Table 4). Combined with the results described above, this finding would appear to replicate several studies implicating a profound decrease in CeA cAMP/protein kinase A (PKA) activity in the context of alcohol dependence (Repunte-Canonigo et al., 2007), a result that may also extend to other extended amygdala regions (Misra and Pandey, 2006), across species (Fitzpatrick-Schmidt et al., 2025), and in relation to additional PDE isoforms (Logrip, 2015). Indeed, a recent therapeutic strategy for inhibiting multiple PDEs with ibudilast has shown considerable promise in preclinical animal models of dependence (Bell et al., 2015). Unfortunately, ibudilast was unable to significantly reduce drinking in a recent phase 2 clinical trial in treatment-seeking individuals with AUD (Ray et al., 2025). Our current findings suggest that mifepristone may represent an alternative mechanistic route to reduce PDE10A activity and resultant alcohol misuse.

### 4.3. Female-specific changes in the serotonin system

In comparison to males, adult female Wistar rats displayed distinct proteomic responses to alcohol. For example, CIEV reduced levels of tryptophan 5-hydroxylase 2 (TPH2; Log2FC = −0.53; Supplemental Table 6), the rate-limiting enzyme in serotonin synthesis, exclusively in females. Given serotonin’s critical role in mood regulation, this result may correspond to the higher prevalence of depression among females with AUD (Moncrieff et al., 2023; McHugh, 2019). Alcohol-induced activation of microglia in the serotonergic dorsal raphe nucleus (DRN) was found to decrease serotonin levels in amygdala terminals in association with increased nociceptive sensitivity and depression-like behaviors (Khan et al., 2023). In the current study, mifepristone did not normalize alcohol-induced reductions in TPH2, pointing to a possible divergence in treatment strategy across sexes. These findings raise the possibility of a treatment model where mifepristone may be more suitable for males, while serotonergic agents such as selective serotonin reuptake inhibitors (SSRIs) or emerging psychedelic strategies (Miller et al., 2025) may offer greater therapeutic benefits for females with AUD and comorbid depression symptoms.

### 4.4. Alterations in neuropeptide systems

The CeA contains a variety of stress and anti-stress neuropeptide systems that are dysregulated in the context of alcohol dependence (Delery and Edwards, 2020). We found that CIEV increased prodynorphin in the CeA of male rats (Log2FC = 0.37; Supplemental Table 1). Prodynorphin is a precursor to the opioid dynorphin, and this neuropeptide activates kappa opioid receptors (KORs), which are linked to dysphoria, stress reactivity, and negative affect (Chavkin, 2008; Hang et al., 2015; Land et al., 2008). KOR signaling is thus widely implicated in the pathophysiology of substance use disorders, including stress-induced reinstatement and drug-seeking behavior (Bruchas et al., 2010; Chavkin and Koob, 2016; Karkhanis et al., 2017; Leconte et al., 2022). A potentiation of dynorphin-KOR systems in the context of alcohol dependence has been described across multiple preclinical models of AUD (Walker and Koob, 2008), including functional neuroadaptations within the CeA (Kissler et al., 2014; Anderson et al., 2019; Haun et al., 2022). Interestingly, mifepristone did not reverse the upregulation of prodynorphin observed in our current study, highlighting the potential independence of these two stress-regulatory systems. This suggests the possibility of targeting the dynorphin-KOR system in parallel with glucocorticoid antagonism for more comprehensive treatment of AUD strategies, or more selective targeting of either system for distinct AUD patient populations.

In parallel, CIEV also increased levels of protachykinin-1 (Log2FC = 0.59; Supplemental Table 1), the precursor to the tachykinin peptide hormone family that includes substance P. This family of peptides is known to mediate neurogenic inflammation, nociception, and anxiety-like behavior (Ebner and Singewald, 2006; Humes et al., 2024; O’Connor et al., 2004; Zieglgänsberger, 2019). Substance P acts via neurokinin-1 (NK1) receptors in the amygdala to modulate nociceptive signaling and stress responses (Linnman et al., 2010; Gadd et al., 2003; Hoppe et al., 2018). Our results also support prior evidence of a functional potentiation of substance P/NK1 signaling in the CeA of both alcohol-preferring and alcohol-dependent rats (Nelson et al., 2019; Khom et al., 2020). Importantly, mifepristone treatment reversed the alcohol-induced increase in protachykinin-1 levels (Log2FC = −0.62; Supplemental Table 4), suggesting that mifepristone’s efficacy may, in part, result from the modification of substance P/NK1 activity, further underscoring the promising role of this neuropeptide system as a viable target for AUD treatment (Schank and Heilig, 2017).

Lastly, alcohol exposure increased cholecystokinin (CCK) expression in females (Log2FC = 0.98; Supplemental Table 6), CCK is the most abundant neuropeptide in mammalian brains and is associated with anxiety, satiety, and affect regulation (Daugé and Léna, 1998; Rotzinger and Vaccarino, 2003; Peikin, 1989), and mifepristone reversed this effect (Log2FC = −1.06; Supplemental Table 10). Interestingly, CCK is known to interact with orexin systems among others, modulating alcohol consumption and satiety (Ballaz et al., 2021). Indeed, this system has been widely implicated across multiple preclinical addiction models and also appears to be increased by estrogen activity within the brain (Ma and Giardino, 2022). The normalization of CCK expression with mifepristone suggests another potential avenue for understanding sex-specific systems and symptoms regulated by neuropeptides that manifest in the dependent state.

### 4.5. Neurometabolic and bioenergetic adaptations

Glucocorticoids were originally named based on their ability to ensure proper energy substrate availability in the context of the systemic stress response, and these critical functions likely extend to the neuronal regulation of energy and nutrient balance. Notably, mifepristone treatment in the context of CIEV upregulated docosahexaenoic acid (DHA) signaling in females (activation z-score = 2; Fig. 7B; Supplemental Table 10). DHA, an important omega-3 fatty acid, supports neuronal membrane integrity, resolution of neuroinflammation, and synaptic plasticity (Farooqui, 2009; Calder, 2013; Banaszak et al., 2024; Tanaka et al., 2012; Cao et al., 2009). These effects suggest that mifepristone may confer bioenergetic and anti-inflammatory benefits, particularly relevant to the female CeA dysregulated by chronic alcohol exposure. Indeed, studies have shown potentially protective effects of DHA in the context of alcohol-induced neuronal insults and resultant behavioral consequences (Feltham et al., 2020; Galduróz et al., 2020; Cardona et al., 2025).

Additionally, alcohol exposure altered levels of glycerol-3-phosphate dehydrogenase 1 (GPD1), a key enzyme in glucose and lipid metabolism, energy production, and mitochondrial redox balance (Oh et al., 2024; Yeh et al., 2008; Mráček et al., 2013). In males, GPD1 was upregulated by CIEV (Log2FC = 0.53; Supplemental Table 1) and normalized by mifepristone (Log2FC = −0.37; Supplemental Table 4), while the opposite pattern was seen in females (Log2FC = −0.44; Supplemental Table 6; Log2FC = 0.52; Supplemental Table 10, respectively). These sex-specific changes in brain bioenergetic systems may further inform tailored pharmacological and/or nutritional approaches for AUD.

**Fig. 9.**
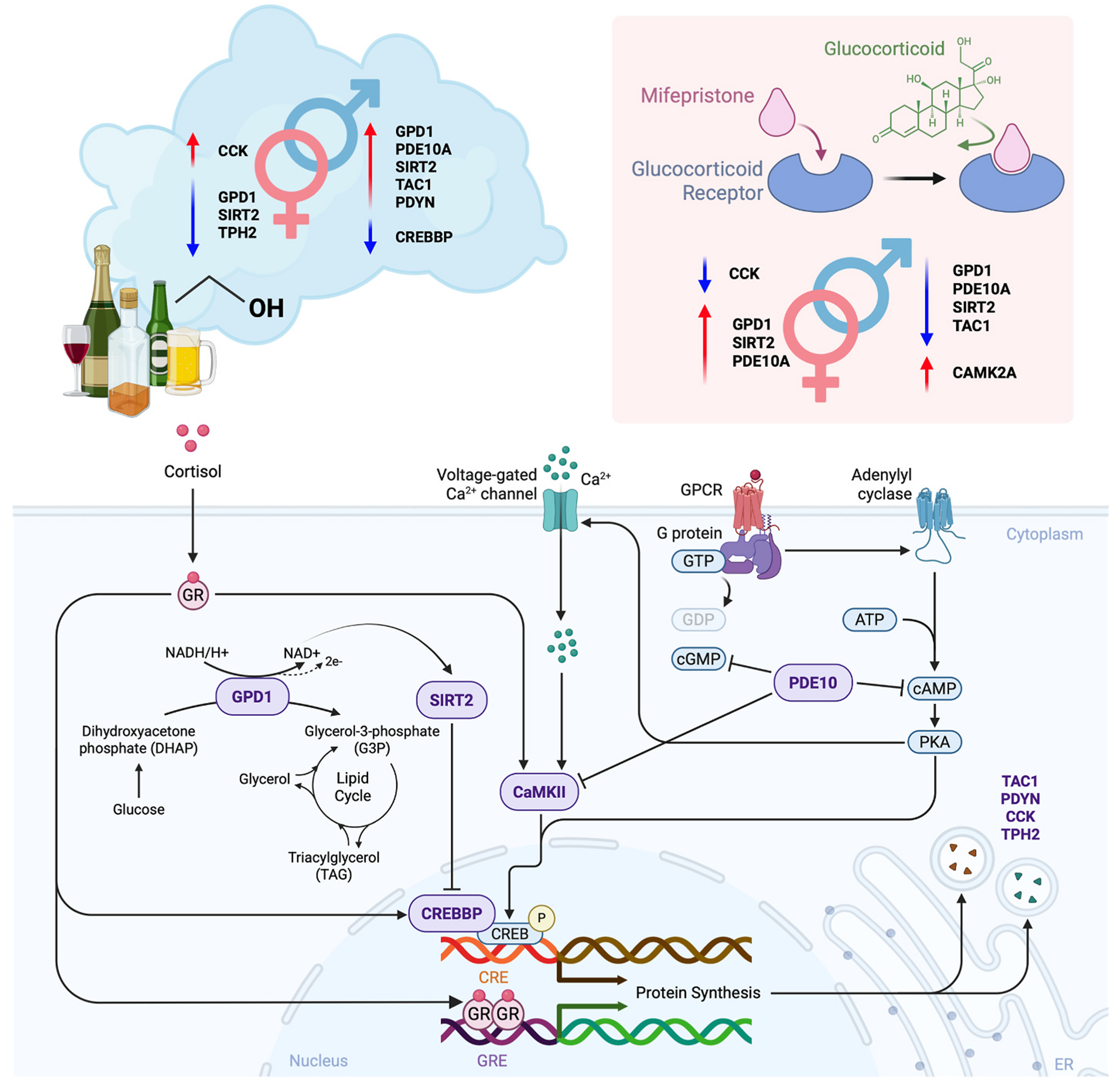
Graphical Summary of Proteins and Pathways Impacted by CIEV and Mifepristone Treatment in Male and Female Wistar Rat Central Amygdala. CIEV upregulates CCK and downregulates GPD1, SIRT2, and TPH2 in female rats. CIEV upregulates GPD1, PDE10A, SIRT2, TAC1, and PDYN and downregulates CREBBP in male rats. Mifepristone downregulates CCK and upregulates GPD1, SIRT2, and PDE10A in female rats. Mifepristone downregulates GPD1, PDE10A, SIRT2, and TAC1 and upregulates CAMK2A in male rats. CCK: cholecystokinin, GPD1: glycerol-3-phosphate dehydrogenase 1, TPH2: tryptophan hydroxylase 2, PDE10A: phosphodiesterase 10A, TAC1: tachykinin 1, PDYN: prodynorphin, CREBBP: CREB binding protein, CAMK2A: calcium/calmodulin-dependent protein kinase 2A. Created in BioRender.

We also discovered significant sex-specific regulation of AMP-activated kinase (AMPK) and sirtuin pathways, which are crucial cellular sensors of energy status and metabolic stress (Sharma et al., 2023; Garcia and Shaw, 2017). In males, chronic alcohol exposure suppressed AMPK signaling (activation z-score = −2.53; Supplemental Table 5) and upregulated sirtuin expression (Log2FC = 0.75; Supplemental Table 1), both of which were normalized by mifepristone. The opposite trend was observed in females. The AMPK and sirtuins work together to not only regulate mitochondrial function, such as bioenergetics and metabolic homeostasis, but also intersect with inflammatory signaling (Elesela et al., 2020; Mihaylova and Shaw, 2011; Ruderman et al., 2010), reinforcing the theme of disrupted bioenergetics and inflammation in the CeA during alcohol dependence. Several of the proteins and pathways we have highlighted are intricately related, and Fig. 9 summarizes key pathways altered by alcohol and mifepristone.

### 4.6. Study limitations and conclusions

One potential limitation of the present study design is the use of chronic mifepristone administration over a three-week period, which raises the possibility of pharmacological tolerance to treatment. However, our dosing regimen was selected based on prior studies demonstrating sustained efficacy of pellet-based extended release in preventing the escalation of alcohol self-administration and stress-related behaviors in rodents (Somkuwar et al., 2017; Vendruscolo et al., 2012) as well as the effects of chronic mifepristone administration to reduce alcohol seeking and craving in humans (Haass-Koffler et al., 2023; Vendruscolo et al., 2015). While our design focused on the determination of neuroadaptations under continuous mifepristone treatment conditions, future work should assess which alterations persist after termination of mifepristone therapy. This design also did not permit the elucidation of genomic vs. non-genomic GR signaling contributions to our discoveries, representing another important aim of future work. Additionally, euthanasia at 6 h withdrawal was chosen to coincide with the peak emergence of withdrawal-related negative affective states in both sexes, ensuring that proteomic changes reflect a behavioral phenotype consistent with previous studies.

Mifepristone’s ability to reduce alcohol self-administration in preclinical animal models extends to more selective GR antagonists and modifiers (McGinn et al., 2021). Indeed, different GR modulators recruit distinct GR-associated transcriptional cofactors and may thereby confer unique neurobiological and systemic safety and efficacy profiles. For the current study, we chose to focus on mifepristone based on its FDA-approved status and proven efficacy in humans. We base our mechanistic interpretations largely on its ability to alter glucocorticoid system activity. However, while the neuroprotective and anti-stress-related effects of mifepristone are largely attributed to its ability to reduce GR transcriptional activity functionally, mifepristone is, in fact, a mixed antagonist at both glucocorticoid and progesterone receptors. Thus, results from this work should be interpreted in this cautionary light, and future experimental designs employing more selective GR antagonists could be valuable. In addition, recent clinical evidence suggests that mifepristone may be particularly effective in reducing stress-associated alcohol craving (Haass-Koffler et al., 2023). Additional studies of mifepristone-induced neuroadaptations in brain regions associated with the anticipation/preoccupation stage of addiction are warranted, including prefrontal cortical areas where GR signaling is activated in the context of alcohol withdrawal (McGinn et al., 2020).

Taken together, new discoveries in the current research study offer valuable insights into sex-specific CeA adaptations to chronic alcohol exposure and putative mifepristone therapy for individuals suffering from AUD. In males, mifepristone reversed many of the CIEV-induced alterations in signaling pathways related to CREB, CaMKII, AMPK, PDE10A, and stress and inflammatory mediators. In females, mifepristone effects were observed in pathways involving DHA signaling, lipid metabolism, and CCK modulation. Notably, several alcohol-induced molecular changes, such as increases in dynorphin signaling in males and reductions in serotonin biosynthetic enzymes in females, were not reversed by mifepristone, highlighting potential differences in treatment responsiveness across sexes. These findings not only strengthen the rationale for targeting glucocorticoid signaling in AUD but also support the emerging need to tailor pharmacotherapeutic strategies to the unique neurobiological profiles of males and females. As we move toward a more mechanistic and individualized approach to pharmacotherapy, understanding the sexually dimorphic effects of chronic alcohol exposure and stress hormone modulation will be vital to the development of more effective and targeted interventions for AUD and related co-morbid conditions.

## Supplementary Material

1

## Figures and Tables

**Table 1:** Description of group comparisons and interpretive results. Air-Placebo (n = 6/sex); Air-Mifepristone (n = 6/sex); CIEV-Placebo (n = 4/sex); CIEV-Mifepristone (n = 4/sex). CIEV, chronic intermittent ethanol vapor.

Group Comparison	Result
CIEV-Placebo vs. Air-Placebo	CIEV effects
Air-Mifepristone vs. Air-Placebo	Mifepristone effects
CIEV-Mifepristone vs. Air-Mifepristone	CIEV effects in the context of mifepristone
CIEV-Mifepristone vs. CIEV-Placebo	Mifepristone effects in the context of CIEV

## Appendix A. Supplementary data

Supplementary data to this article can be found online at https://doi.org/10.1016/j.neuropharm.2025.110728.
