# Supplementary material for: Proteomic analysis of central amygdala systems regulated by mifepristone in the context of alcohol dependence
Source: Neuropharmacology. Author manuscript; Available in PMC 2026 Jun 24. (PMC13293126; doi:10.1016/j.neuropharm.2025.110728)
Supplement: 1 [file NIHMS2184984-supplement-1.docx]

| CIEV-Placebo vs. Air-Placebo | | | |
| --- | --- | --- | --- |
|  | **Gene**  **Symbol** | **Description** | **Log2FC** |
| Downregulated | Sphkap | A-kinase anchor protein SPHKAP | -3.61 |
|  | Arhgef1 | Rho guanine nucleotide exchange factor 1 | -1.96 |
|  | Crcp | DNA-directed RNA polymerase III subunit RPC9 | -1.95 |
|  | Timmdc1 | Complex I assembly factor TIMMDC1, mitochondrial | -1.69 |
|  | Magmas-ps1 | Mitochondrial import inner membrane translocase subunit TIM16 | -1.66 |
|  | Pik3r2 | Phosphatidylinositol 3-kinase regulatory subunit beta | -1.54 |
|  | Unc5a | Netrin receptor UNC5A | -1.52 |
|  | Mapk14 | Mitogen-activated protein kinase 14 | -1.49 |
|  | Nras | GTPase NRas | -1.38 |
|  | Ighg; Ighg1 | Ig gamma-1 chain C region | -1.34 |
|  | Set | Isoform 2 of Protein SET | -1.31 |
|  | Ncbp1 | Nuclear cap-binding protein subunit 1 | -1.26 |
|  | Morn4 | MORN repeat-containing protein 4 | -1.22 |
|  | Anks1b | Isoform 3 of Ankyrin repeat and sterile alpha motif domain-containing protein 1B | -1.15 |
|  | Hacl1 | 2-hydroxyacyl-CoA lyase 1 | -1.15 |
|  | Kctd5 | BTB/POZ domain-containing protein KCTD5 | -1.15 |
|  | Os9 | Protein os-9 | -1.1 |
|  | Cacng2 | Voltage-dependent calcium channel gamma-2 subunit | -1.08 |
|  | Slc22a4 | Solute carrier family 22 member 4 | -1.07 |
|  | Kcnj3 | G protein-activated inward rectifier potassium channel 1 | -1.05 |
|  | Crebbp | CREB-binding protein | -1.05 |
|  | Gria1 | Glutamate receptor 1 | -1.03 |
|  | Elp6 | Elongator complex protein 6 | -1.02 |
|  | Slc17a7 | Vesicular glutamate transporter 1 | -0.96 |
|  | Kalrn | Kalirin | -0.91 |
|  | Gria2 | Isoform Flip of Glutamate receptor 2 | -0.9 |
|  | Gabrg3 | Gamma-aminobutyric acid receptor subunit gamma-3 | -0.89 |
|  | Taco1 | Translational activator of cytochrome c oxidase 1 | -0.86 |
|  | Necab1 | N-terminal EF-hand calcium-binding protein 1 | -0.85 |
|  | Sv2b | Synaptic vesicle glycoprotein 2B | -0.85 |
|  | Gfpt1 | Glutamine-fructose-6-phosphate aminotransferase [isomerizing] 1 | -0.85 |
|  | ND2 | NADH-ubiquinone oxidoreductase chain 2 | -0.83 |
|  | Hpcal1 | Hippocalcin-like protein 1 | -0.81 |
|  | Sub1 | Activated RNA polymerase II transcriptional coactivator p15 | -0.8 |
|  | Ncan | Neurocan core protein | -0.79 |
|  | Aktip | AKT-interacting protein | -0.79 |
|  | Oxr1 | Isoform 2 of Oxidation resistance protein 1 | -0.79 |
|  | Dcps | M7GpppX diphosphatase | -0.76 |
|  | Atp4a | Potassium-transporting ATPase alpha chain 1 | -0.74 |
|  | Vwa5a; LOC108348048 | von Willebrand factor A domain-containing protein 5A | -0.73 |
|  | Nlgn1 | Neuroligin-1 | -0.72 |
|  | Abca2 | ATP-binding cassette sub-family A member 2 | -0.71 |
|  | Nif3l1 | NIF3-like protein 1 | -0.71 |
|  | Eif4a3 | Eukaryotic initiation factor 4A-III | -0.7 |
|  | Gabra2 | Gamma-aminobutyric acid receptor subunit alpha-2 | -0.7 |
|  | Chrm1 | Muscarinic acetylcholine receptor M1 | -0.69 |
|  | Prepl | prolyl endopeptidase-like | -0.68 |
|  | Faah; LOC100911581 | Fatty-acid amide hydrolase 1 | -0.68 |
|  | Grm2 | Metabotropic glutamate receptor 2 | -0.68 |
|  | Ss18l1 | Calcium-responsive transcription coactivator | -0.68 |
|  | Olfm2 | Noelin-2 | -0.68 |
|  | Ssrp1 | FACT complex subunit SSRP1 | -0.68 |
|  | Nos1 | Nitric oxide synthase, brain | -0.68 |
|  | Vezt | vezatin | -0.67 |
|  | Cd59 | CD59 glycoprotein | -0.66 |
|  | Hapln1 | Hyaluronan and proteoglycan link protein 1 | -0.66 |
|  | Sfxn1 | Sideroflexin-1 | -0.66 |
|  | Rtn1 | Isoform RTN1-S of Reticulon-1 | -0.66 |
|  | Gnpat | Dihydroxyacetone phosphate acyltransferase | -0.66 |
|  | Mpp2 | MAGUK p55 subfamily member 2 | -0.65 |
|  | Timp2 | Metalloproteinase inhibitor 2 | -0.65 |
|  | Gpr56; Adgrg1 | Adhesion G-protein coupled receptor G1 | -0.65 |
|  | Syngr1 | Synaptogyrin-1 | -0.64 |
|  | Glrx | Glutaredoxin-1 | -0.64 |
|  | Hpcal4 | Hippocalcin-like protein 4 | -0.63 |
|  | Cnrip1 | CB1 cannabinoid receptor-interacting protein 1 | -0.63 |
|  | Grin2b | Glutamate receptor ionotropic, NMDA 2B | -0.62 |
|  | Hmgcs1 | hydroxymethylglutaryl-CoA synthase, cytoplasmic | -0.61 |
|  | Cpne7 | Copine-7 | -0.61 |
|  | Ilf2 | Interleukin enhancer-binding factor 2 | -0.6 |
|  | Rab3c | ras-related protein Rab-3C | -0.6 |
|  | Dlgap3 | Disks large-associated protein 3 | -0.6 |
|  | Pnp | purine nucleoside phosphorylase | -0.6 |
|  | Ptbp2 | Polypyrimidine tract-binding protein 2 | -0.6 |
|  | Vcam1 | Vascular cell adhesion protein 1 | -0.6 |
|  | LOC100362027; Rpl30 | 60S ribosomal protein L30 | -0.59 |
|  | Tomm34 | Mitochondrial import receptor subunit TOM34 | -0.59 |
|  | Rab27a | Ras-related protein Rab-27A | -0.59 |
|  | Ssbp3 | Single-stranded DNA-binding protein 3 | -0.59 |
|  | Prkar2a | cAMP-dependent protein kinase type II-alpha regulatory subunit | -0.58 |
|  | Nptxr | neuronal pentraxin receptor | -0.58 |
|  | Gaa | lysosomal alpha-glucosidase | -0.58 |
|  | Grm3 | Metabotropic glutamate receptor 3 | -0.57 |
|  | Olfm1 | Noelin | -0.57 |
|  | Dclk1 | serine/threonine-protein kinase DCLK1 | -0.56 |
|  | Pcyox1 | Prenylcysteine oxidase | -0.56 |
|  | Hnrnpa1 | Heterogeneous nuclear ribonucleoprotein A1 | -0.56 |
|  | Dgkb | Diacylglycerol kinase beta | -0.56 |
|  | Hnrpd; Hnrnpd | heterogeneous nuclear ribonucleoprotein D0 | -0.56 |
|  | Dlg4 | Disks large homolog 4 | -0.55 |
|  | Eif4a2 | eukaryotic initiation factor 4A-II | -0.55 |
|  | Cand2 | Cullin-associated NEDD8-dissociated protein 2 | -0.55 |
|  | Calb2 | Calretinin | -0.55 |
|  | Grin1 | Glutamate receptor ionotropic, NMDA 1 | -0.54 |
|  | Prkra | Interferon-inducible double-stranded RNA-dependent protein kinase activator A | -0.54 |
|  | Chrm4 | Muscarinic acetylcholine receptor M4 | -0.54 |
|  | Limk1 | LIM domain kinase 1 | -0.54 |
|  | Hnrnpa3 | Heterogeneous nuclear ribonucleoprotein A3 | -0.53 |
|  | Itpka | Inositol-trisphosphate 3-kinase A | -0.53 |
|  | Puf60 | poly(U)-binding-splicing factor PUF60 | -0.53 |
|  | Ncald | Neurocalcin-delta | -0.52 |
|  | Pafah1b3 | Platelet-activating factor acetylhydrolase IB subunit gamma | -0.52 |
|  | Pnck | Calcium/calmodulin-dependent protein kinase type 1B | -0.52 |
|  | Ptk2b | Protein-tyrosine kinase 2-beta | -0.52 |
|  | Ctsz | Cathepsin Z | -0.51 |
|  | Pspc1 | Paraspeckle component 1 | -0.51 |
|  | LOC100360449; Rpl9; LOC100364457 | 60S ribosomal protein L9 | -0.5 |
|  | Slc12a5 | Solute carrier family 12 member 5 | -0.5 |
|  | Ilf3 | Interleukin enhancer-binding factor 3 | -0.5 |
|  | Ezr | Ezrin | -0.5 |
|  | Dnajc16 | DnaJ homolog subfamily C member 16 | -0.5 |
|  | Gnaz | guanine nucleotide-binding protein G(z) subunit alpha | -0.49 |
|  | Cc2d1a | Coiled-coil and C2 domain-containing protein 1A | -0.49 |
|  | Matr3 | Matrin-3 | -0.49 |
|  | Usp46 | Ubiquitin carboxyl-terminal hydrolase 46 | -0.49 |
|  | Prkacb | cAMP-dependent protein kinase catalytic subunit beta | -0.49 |
|  | Slc4a10 | Sodium-driven chloride bicarbonate exchanger | -0.49 |
|  | Tp53i11 | Tumor protein p53-inducible protein 11 | -0.48 |
|  | LOC100362640; Rps4x; Rps4x-ps9 | 40S ribosomal protein S4, X isoform | -0.47 |
|  | LOC687090; Tprg1l | Tumor protein p63-regulated gene 1-like protein | -0.47 |
|  | Rpl15 | 60S ribosomal protein L15 | -0.47 |
|  | Hnrnpa2b1 | heterogeneous nuclear ribonucleoproteins A2/B1 | -0.46 |
|  | Maoa | Amine oxidase [flavin-containing] A | -0.46 |
|  | Elavl1 | ELAV-like protein 1 | -0.46 |
|  | Ptprs | Receptor-type tyrosine-protein phosphatase S | -0.46 |
|  | Synpo | synaptopodin | -0.46 |
|  | Lrpap1 | alpha-2-macroglobulin receptor-associated protein | -0.46 |
|  | Lphn3; Adgrl3 | Adhesion G protein-coupled receptor L3 | -0.46 |
|  | Ncs1 | neuronal calcium sensor 1 | -0.45 |
|  | Acot7 | Isoform 1 of Cytosolic acyl coenzyme A thioester hydrolase | -0.45 |
|  | Tagln3; LOC103693564 | Transgelin-3 | -0.44 |
|  | Atp2b4 | Isoform XA of Plasma membrane calcium-transporting ATPase 4 | -0.44 |
|  | Sh3gl2 | Endophilin-A1 | -0.44 |
|  | Plxna3 | Plexin-A3 | -0.44 |
|  | Rnase4 | Ribonuclease 4 | -0.44 |
|  | Ocrl | Inositol polyphosphate 5-phosphatase OCRL-1 | -0.44 |
|  | Syn2 | Synapsin-2 | -0.43 |
|  | Sae1 | SUMO-activating enzyme subunit 1 | -0.43 |
|  | Eif3h; LOC100911110; LOC108348062 | Eukaryotic translation initiation factor 3 subunit H | -0.43 |
|  | Tmed2 | Transmembrane emp24 domain-containing protein 2 | -0.43 |
|  | Ddx39b | spliceosome RNA helicase DDX39B | -0.43 |
|  | Ecsit | Evolutionarily conserved signaling intermediate in Toll pathway, mitochondrial | -0.43 |
|  | Snrpb | small nuclear ribonucleoprotein-associated protein B | -0.43 |
|  | Pi4ka | phosphatidylinositol 4-kinase alpha | -0.42 |
|  | Slc1a3 | Excitatory amino acid transporter 1 | -0.42 |
|  | Adrbk1; Grk2 | Beta-adrenergic receptor kinase 1 | -0.42 |
|  | Actn1 | Alpha-actinin-1 | -0.42 |
|  | Flot2 | Flotillin-2 | -0.42 |
|  | Copb1 | Coatomer subunit beta | -0.42 |
|  | Ppp1cb | Serine/threonine-protein phosphatase PP1-beta catalytic subunit | -0.42 |
|  | Hspbp1 | Hsp70-binding protein 1 | -0.42 |
|  | Macf1 | Microtubule-actin cross-linking factor 1 | -0.41 |
|  | Slc8a1 | Isoform 2 of Sodium/calcium exchanger 1 | -0.41 |
|  | Ptpn9 | Tyrosine-protein phosphatase non-receptor type 9 | -0.41 |
|  | Rps2 | 40S ribosomal protein S2 | -0.41 |
|  | Rps9; LOC100909466; LOC103689992 | 40S ribosomal protein S9 | -0.4 |
|  | Rpsa | 40S ribosomal protein SA | -0.4 |
|  | Crmp1 | Dihydropyrimidinase-related protein 1 | -0.4 |
|  | Rpl14 | 60S ribosomal protein L14 | -0.4 |
|  | Nptx1 | Neuronal pentraxin-1 | -0.4 |
|  | Hsd17b10 | 3-hydroxyacyl-CoA dehydrogenase type-2 | -0.39 |
|  | Hspa13 | Heat shock 70 kDa protein 13 | -0.39 |
|  | Rpn2 | Dolichyl-diphosphooligosaccharide--protein glycosyltransferase subunit 2 | -0.39 |
|  | Gna11 | guanine nucleotide-binding protein subunit alpha-11 | -0.39 |
|  | Ap1b1 | AP-1 complex subunit beta-1 | -0.39 |
|  | Gnb2l1; Rack1; LOC100911540 | Receptor of activated protein C kinase 1 | -0.39 |
|  | Rpl18a | 60S ribosomal protein L18a | -0.39 |
|  | Gda | guanine deaminase | -0.38 |
|  | Arpc2 | Actin-related protein 2/3 complex subunit 2 | -0.38 |
|  | Htt | huntingtin | -0.38 |
|  | Camkv | caM kinase-like vesicle-associated protein | -0.37 |
|  | Nono | Non-POU domain-containing octamer-binding protein | -0.37 |
|  | Poglut1 | Protein O-glucosyltransferase 1 | -0.37 |
|  | Pfkl | ATP-dependent 6-phosphofructokinase, liver type | -0.36 |
|  | COX2 | Cytochrome c oxidase subunit 2 | -0.36 |
|  | Sec14l2 | SEC14-like protein 2 | -0.36 |
|  | Prkar2b | cAMP-dependent protein kinase type II-beta regulatory subunit | -0.36 |
|  | Ptprn | Receptor-type tyrosine-protein phosphatase-like N | -0.36 |
|  | Cotl1 | coactosin-like protein | -0.36 |
|  | Ap2b1; LOC100912146 | AP-2 complex subunit beta | -0.35 |
|  | Gstz1 | maleylacetoacetate isomerase | -0.35 |
|  | Coro1a | Coronin-1A | -0.35 |
|  | Hap1 | Huntingtin-associated protein 1 | -0.35 |
|  | Nisch | Nischarin | -0.35 |
|  | Lrpprc | Leucine-rich PPR motif-containing protein, mitochondrial | -0.35 |
|  | B2m | Beta-2-microglobulin | -0.35 |
|  | Gmps | GMP synthase [glutamine-hydrolyzing] | -0.35 |
|  | Otub1 | Ubiquitin thioesterase otub1 | -0.34 |
|  | Rpl11 | 60S ribosomal protein L11 | -0.34 |
|  | Anp32a | Acidic leucine-rich nuclear phosphoprotein 32 family member A | -0.34 |
|  | Ak1 | Adenylate kinase isoenzyme 1 | -0.34 |
|  | Shank3 | Isoform 1 of SH3 and multiple ankyrin repeat domains protein 3 | -0.34 |
|  | Txnl1 | Thioredoxin-like protein 1 | -0.34 |
|  | Prps1; LOC314140 | ribose-phosphate pyrophosphokinase 1 | -0.34 |
|  | Nlrx1 | NLR family member X1 | -0.34 |
|  | Rpl26 | 60S ribosomal protein L26 | -0.34 |
|  | Eif3a | Eukaryotic translation initiation factor 3 subunit A | -0.34 |
|  | Cltc | Clathrin heavy chain 1 | -0.33 |
|  | Ddb1 | DNA damage-binding protein 1 | -0.33 |
|  | Eef1a2 | Elongation factor 1-alpha 2 | -0.33 |
|  | Cyld | Ubiquitin carboxyl-terminal hydrolase CYLD | -0.33 |
| Upregulated | Kcna2 | Potassium voltage-gated channel subfamily A member 2 | 0.33 |
|  | Atp5i | ATP synthase subunit e, mitochondrial | 0.33 |
|  | Rac1 | Ras-related C3 botulinum toxin substrate 1 | 0.35 |
|  | Eml1 | Echinoderm microtubule-associated protein-like 1 | 0.36 |
|  | Cntn2 | Contactin-2 | 0.36 |
|  | Tbca | Tubulin-specific chaperone A | 0.37 |
|  | Pdyn | Prodynorphin | 0.37 |
|  | Gap43 | Neuromodulin | 0.38 |
|  | Gsta3; Gsta1; LOC102550391; LOC108348061 | glutathione S-transferase alpha-3 | 0.38 |
|  | Lap3 | cytosol aminopeptidase | 0.4 |
|  | Ca2; Car2 | Carbonic anhydrase 2 | 0.4 |
|  | Ptma; LOC100359583 | Prothymosin alpha | 0.4 |
|  | Slc6a11 | Sodium- and chloride-dependent GABA transporter 3 | 0.43 |
|  | Fah | Fumarylacetoacetase | 0.43 |
|  | Camk2b | Calcium/calmodulin-dependent protein kinase type II subunit beta | 0.45 |
|  | Gstt2 | Glutathione S-transferase theta-2 | 0.47 |
|  | Atp5j | ATP synthase-coupling factor 6, mitochondrial | 0.5 |
|  | Arpp19; LOC100360828 | cAMP-regulated phosphoprotein 19 | 0.5 |
|  | Reep5 | Receptor expression-enhancing protein 5 | 0.51 |
|  | Csrp1 | Cysteine and glycine-rich protein 1 | 0.52 |
|  | Gpd1 | Glycerol-3-phosphate dehydrogenase [NAD(+)], cytoplasmic | 0.53 |
|  | Map1a | Microtubule-associated protein 1A | 0.55 |
|  | Enpp6 | Ectonucleotide pyrophosphatase/phosphodiesterase family member 6 | 0.56 |
|  | Gstp1 | Glutathione S-transferase P | 0.58 |
|  | Qdpr | dihydropteridine reductase | 0.58 |
|  | Wipf3 | WAS/WASL-interacting protein family member 3 | 0.58 |
|  | Tac1 | protachykinin-1 | 0.59 |
|  | Jup | Junction plakoglobin | 0.61 |
|  | Ppp1r2 | Protein phosphatase inhibitor 2 | 0.62 |
|  | Slc44a1 | choline transporter-like protein 1 | 0.66 |
|  | Gsn | Gelsolin | 0.67 |
|  | Nfasc | Isoform 2 of Neurofascin | 0.69 |
|  | Pllp | plasmolipin | 0.69 |
|  | Sirt2 | NAD-dependent protein deacetylase sirtuin-2 | 0.75 |
|  | Cldn11 | Claudin-11 | 0.78 |
|  | Ermn | ermin | 0.8 |
|  | Mog | Myelin-oligodendrocyte glycoprotein | 0.81 |
|  | Cd82 | CD82 antigen | 0.82 |
|  | Myo1d | Unconventional myosin-Id | 0.83 |
|  | Clic4 | Chloride intracellular channel protein 4 | 0.84 |
|  | Ppp1r14a | Protein phosphatase 1 regulatory subunit 14A | 0.86 |
|  | Gng7 | guanine nucleotide-binding protein G(I)/G(S)/G(O) subunit gamma-7 | 0.89 |
|  | Cnp | 2',3'-cyclic-nucleotide 3'-phosphodiesterase | 0.9 |
|  | Mobp | myelin-associated oligodendrocyte basic protein | 0.95 |
|  | Cd151; LOC100911730 | CD151 antigen | 0.99 |
|  | Tsc22d3 | TSC22 domain family protein 3 | 1.03 |
|  | Krt17 | Keratin, type I cytoskeletal 17 | 1.08 |
|  | Krt10 | Keratin, type I cytoskeletal 10 | 1.11 |
|  | Cd81 | CD81 antigen | 1.12 |
|  | Plp1 | Myelin proteolipid protein | 1.14 |
|  | Gltp | glycolipid transfer protein | 1.14 |
|  | Mbp | myelin basic protein | 1.15 |
|  | Ina | alpha-internexin | 1.2 |
|  | Krt2 | Keratin, type II cytoskeletal 2 epidermal | 1.22 |
|  | Nefl | Neurofilament light polypeptide | 1.24 |
|  | Krt1 | Keratin, type II cytoskeletal 1 | 1.25 |
|  | Hcn3 | Potassium/sodium hyperpolarization-activated cyclic nucleotide-gated channel 3 | 1.26 |
|  | Nefm | Neurofilament medium polypeptide | 1.29 |
|  | Krt5 | keratin, type II cytoskeletal 5 | 1.3 |
|  | Cd9 | CD9 antigen | 1.36 |
|  | Krt72 | Keratin, type II cytoskeletal 72 | 1.42 |
|  | Mag | Myelin-associated glycoprotein | 1.54 |
|  | Nefh | Neurofilament heavy polypeptide | 1.56 |
|  | Krt8 | Keratin, type II cytoskeletal 8 | 1.6 |
|  | Pcp4 | calmodulin regulator protein PCP4 | 1.64 |
|  | Ppp2cb | serine/threonine-protein phosphatase 2A catalytic subunit beta isoform | 1.66 |
|  | Cryab | Alpha-crystallin B chain | 1.7 |
|  | Cox7c | Cytochrome c oxidase subunit 7C, mitochondrial | 1.81 |
|  | Ppp1r1b | Protein phosphatase 1 regulatory subunit 1B | 1.82 |
|  | Mbp | Isoform 3 of Myelin basic protein | 1.85 |
|  | Pde10a | cAMP and cAMP-inhibited cGMP 3',5'-cyclic phosphodiesterase 10A | 2.17 |
|  | Scn4b | Sodium channel subunit beta-4 | 2.5 |
|  | Sde2 | Protein SDE2 homolog | 2.67 |

***Supplemental Table 1*. A comprehensive list of all proteins significantly downregulated or upregulated by CIEV in male Wistar rats.** The gene symbols, protein names, and Log2FC are included in the table. Only those with a *P* ≤ 0.05 and a FC ≥ 1.25/1 or FC ≤ 1/1.25 are listed.

| CIEV-Mifepristone vs. Air-Mifepristone | | | | | | |  |  |
| --- | --- | --- | --- | --- | --- | --- | --- | --- |
|  | **Gene Symbol** | | **Description** | | **Log2FC** | | |  |
| Downregulated | Cep104 | | Centrosomal protein of 104 kDa | | -1.63 | | |  |
|  | Kng1; Kng2 | | T-kininogen 1 | | -1.49 | | |  |
|  | Scn1a | | Sodium channel protein type 1 subunit alpha | | -1.02 | | |  |
|  | Os9 | | Protein os-9 | | -0.87 | | |  |
|  | Ambp | | Protein AMBP | | -0.86 | | |  |
|  | LOC299282 | | Serine protease inhibitor A3L | | -0.85 | | |  |
|  | Ttr | | Transthyretin | | -0.82 | | |  |
|  | Aldh3b1 | | Aldehyde dehydrogenase family 3 member B1 | | -0.81 | | |  |
|  | A1i3; LOC297568 | | Alpha-1-inhibitor 3 | | -0.75 | | |  |
|  | Serpina3c; Serpina3k | | Serine protease inhibitor A3K | | -0.66 | | |  |
|  | Cdc42se2 | | CDC42 small effector protein 2 | | -0.65 | | |  |
|  | LOC10013487; LOC689064; LOC103694857 | | Hemoglobin subunit beta-2 | | -0.64 | | |  |
|  | Pzp | | Alpha-1-macroglobulin | | -0.64 | | |  |
|  | Ces1c | | Carboxylesterase 1C | | -0.63 | | |  |
|  | Slc4a1 | | Band 3 anion transport protein | | -0.62 | | |  |
|  | C3 | | Complement C3 | | -0.62 | | |  |
|  | Manba | | Beta-mannosidase | | -0.58 | | |  |
|  | Cldn11 | | Claudin-11 | | -0.56 | | |  |
|  | Apoa1 | | Apolipoprotein A-I | | -0.55 | | |  |
|  | Mag | | Myelin-associated glycoprotein | | -0.55 | | |  |
|  | Padi2 | | Protein-arginine deiminase type-2 | | -0.54 | | |  |
|  | Myo1d | | Unconventional myosin-Id | | -0.53 | | |  |
|  | Nefl | | Neurofilament light polypeptide | | -0.53 | | |  |
|  | Ugt8 | | 2-hydroxyacylsphingosine 1-beta-galactosyltransferase | | -0.52 | | |  |
|  | Plp1 | | Myelin proteolipid protein | | -0.50 | | |  |
|  | Nefm | | Neurofilament medium polypeptide | | -0.50 | | |  |
|  | Nefh | | Neurofilament heavy polypeptide | | -0.48 | | |  |
|  | Serpina1 | | Alpha-1-antiproteinase | | -0.47 | | |  |
|  | Ca2; Car2 | | Carbonic anhydrase 2 | | -0.45 | | |  |
|  | Mobp | | myelin-associated oligodendrocyte basic protein | | -0.45 | | |  |
|  | Gltp | | glycolipid transfer protein | | -0.41 | | |  |
|  | Cd82 | | CD82 antigen | | -0.39 | | |  |
|  | Cnp | | 2',3'-cyclic-nucleotide 3'-phosphodiesterase | | -0.39 | | |  |
|  | Slc44a1 | | choline transporter-like protein 1 | | -0.38 | | |  |
|  | Cd9 | | CD9 antigen | | -0.37 | | |  |
|  | Fah | | Fumarylacetoacetase | | -0.36 | | |  |
|  | Qdpr | | dihydropteridine reductase | | -0.35 | | |  |
|  | Lap3 | | cytosol aminopeptidase | | -0.35 | | |  |
|  | Enpp6 | | Ectonucleotide pyrophosphatase/phosphodiesterase family member 6 | | -0.34 | | |  |
|  | Tf | | Serotransferrin | | -0.34 | | |  |
|  | Gfap | | Glial fibrillary acidic protein | | -0.34 | | |  |
|  | Ppp1r14a | | Protein phosphatase 1 regulatory subunit 14A | | -0.33 | | |  |
| Upregulated | | Msh5 | | mutS protein homolog 5 | | 0.66 | | |
|  |  | Rab3a | | Ras-related protein Rab-3A | | 0.70 | | |
|  |  | Gnb2 | | Guanine nucleotide-binding protein G(I)/G(S)/G(T) subunit beta-2 | | 0.99 | | |

***Supplemental Table 2*. A comprehensive list of all proteins significantly downregulated or upregulated by CIEV in the context of mifepristone in male Wistar rats.** The gene symbols, protein names, and Log2FC are included in the table. Only those with a *P* ≤ 0.05 and a FC ≥ 1.25/1 or FC ≤ 1/1.25 are listed.

| Air-Mifepristone vs. Air-Placebo | | | |
| --- | --- | --- | --- |
|  | **Gene Symbol** | **Description** | **Log2FC** |
| Downregulated | Hacl1 | 2-hydroxyacyl-CoA lyase 1 | -0.85 |
|  | Taco1 | Translational activator of cytochrome c oxidase 1 | -0.65 |
|  | Anks1b | Isoform 3 of Ankyrin repeat and sterile alpha motif domain-containing protein 1B | -0.58 |
|  | Dlgap3 | Disks large-associated protein 3 | -0.42 |
|  | Hspbp1 | Hsp70-binding protein 1 | -0.37 |
| Upregulated | Fah | Fumarylacetoacetase | 0.38 |
|  | Gsn | Gelsolin | 0.38 |
|  | Ca2; Car2 | Carbonic anhydrase 2 | 0.39 |
|  | Mobp | myelin-associated oligodendrocyte basic protein | 0.43 |
|  | Manba | Beta-mannosidase | 0.44 |
|  | Enpp6 | Ectonucleotide pyrophosphatase/phosphodiesterase family member 6 | 0.45 |
|  | Ugt8 | 2-hydroxyacylsphingosine 1-beta-galactosyltransferase | 0.45 |
|  | Pcp4 | calmodulin regulator protein PCP4 | 0.48 |
|  | Gltp | glycolipid transfer protein | 0.54 |
|  | Myo1d | Unconventional myosin-Id | 0.54 |
|  | Cnp | 2',3'-cyclic-nucleotide 3'-phosphodiesterase | 0.57 |
|  | Ina | alpha-internexin | 0.58 |
|  | Cd9 | CD9 antigen | 0.58 |
|  | Nefl | Neurofilament light polypeptide | 0.6 |
|  | Cd82 | CD82 antigen | 0.64 |
|  | Abcb9 | ATP-binding cassette sub-family B member 9 | 0.64 |
|  | Cryab | Alpha-crystallin B chain | 0.65 |
|  | Plp1 | Myelin proteolipid protein | 0.67 |
|  | Nefm | Neurofilament medium polypeptide | 0.71 |
|  | Nefh | Neurofilament heavy polypeptide | 0.72 |
|  | Mbp | myelin basic protein | 0.74 |
|  | Mag | Myelin-associated glycoprotein | 0.82 |
|  | Mbp | Isoform 3 of Myelin basic protein | 0.89 |
|  | Pvalb | Parvalbumin alpha | 1.33 |
|  | Pde10a | cAMP and cAMP-inhibited cGMP 3',5'-cyclic phosphodiesterase 10A | 1.4 |

***Supplemental Table 3*. A comprehensive list of all proteins significantly downregulated or upregulated by mifepristone treatment in male Wistar rats.** The gene symbols, protein names, and Log2FC are included in the table. Only those with a *P* ≤ 0.05 and a FC ≥ 1.25/1 or FC ≤ 1/1.25 are listed.

| CIEV-Mifepristone vs. CIEV-Placebo | | | | |
| --- | --- | --- | --- | --- |
|  | **Gene Symbol** | **Description** | **Log2FC** | |
| Downregulated | Sde2 | Protein SDE2 homolog | -3.07 | |
|  | Cox7c | Cytochrome c oxidase subunit 7C, mitochondrial | -2.04 | |
|  | Mbp | Isoform 3 of Myelin basic protein | -1.7 | |
|  | Krt8 | Keratin, type II cytoskeletal 8 | -1.55 | |
|  | Camk2d | Isoform Delta 4 of Calcium/calmodulin-dependent protein kinase type II subunit delta | -1.54 | |
|  | Pcp4 | calmodulin regulator protein PCP4 | -1.42 | |
|  | Scn4b | Sodium channel subunit beta-4 | -1.41 | |
|  | Fgfr1 | Fibroblast growth factor receptor 1 | -1.38 | |
|  | Nefh | Neurofilament heavy polypeptide | -1.32 | |
|  | Pde10a | cAMP and cAMP-inhibited cGMP 3',5'-cyclic phosphodiesterase 10A | -1.31 | |
|  | Ppp1r1b | Protein phosphatase 1 regulatory subunit 1B | -1.3 | |
|  | Mag | Myelin-associated glycoprotein | -1.27 | |
|  | Ppp2cb | serine/threonine-protein phosphatase 2A catalytic subunit beta isoform | -1.27 | |
|  | Nefl | Neurofilament light polypeptide | -1.17 | |
|  | Cd9 | CD9 antigen | -1.15 | |
|  | Nefm | Neurofilament medium polypeptide | -1.08 | |
|  |  | Isoform 5 of Breast carcinoma-amplified sequence 1 homolog | -1.08 | |
|  | Krt5 | keratin, type II cytoskeletal 5 | -1.06 | |
|  | Ermn | ermin | -1.04 | |
|  | LOC683313; LOC100365213 | Keratin, type II cytoskeletal 6A | -1.03 | |
|  | Cd151; LOC100911730 | CD151 antigen | -1.02 | |
|  | Gltp | glycolipid transfer protein | -1.01 | |
|  | Krt1 | Keratin, type II cytoskeletal 1 | -1 | |
|  | Pllp | plasmolipin | -1 | |
|  | Plp1 | Myelin proteolipid protein | -0.97 | |
|  | Mobp | myelin-associated oligodendrocyte basic protein | -0.97 | |
|  | Cryab | Alpha-crystallin B chain | -0.97 | |
|  | Mbp | myelin basic protein | -0.97 | |
|  | Ina | alpha-internexin | -0.94 | |
|  | Krt17 | Keratin, type I cytoskeletal 17 | -0.94 | |
|  | Krt10 | Keratin, type I cytoskeletal 10 | -0.93 | |
|  | Krt2 | Keratin, type II cytoskeletal 2 epidermal | -0.93 | |
|  | Metap2 | methionine aminopeptidase 2 | -0.89 | |
|  | Clic4 | Chloride intracellular channel protein 4 | -0.89 | |
|  | Cdc42se2 | CDC42 small effector protein 2 | -0.88 | |
|  | Ppp1r14a | Protein phosphatase 1 regulatory subunit 14A | -0.87 | |
|  | Fam63a; Mindy1 | Ubiquitin carboxyl-terminal hydrolase MINDY-1 | -0.87 | |
|  | Cldn11 | Claudin-11 | -0.85 | |
|  | LOC299282 | Serine protease inhibitor A3L | -0.85 | |
|  | Lpcat3 | Lysophospholipid acyltransferase 5 | -0.84 | |
|  | Myo1d | Unconventional myosin-Id | -0.83 | |
|  | Apoa1 | Apolipoprotein A-I | -0.8 | |
|  | S100b | Protein S100-B | -0.78 | |
|  | Tpm3 | Tropomyosin alpha-3 chain | -0.76 | |
|  | Cnp | 2',3'-cyclic-nucleotide 3'-phosphodiesterase | -0.72 | |
|  | Ppp1r2 | Protein phosphatase inhibitor 2 | -0.72 | |
|  | Ces1c | Carboxylesterase 1C | -0.7 | |
|  | Slc44a1 | choline transporter-like protein 1 | -0.69 | |
|  | Csrp1 | Cysteine and glycine-rich protein 1 | -0.69 | |
|  | Arpp19; LOC100360828 | cAMP-regulated phosphoprotein 19 | -0.69 | |
|  | Serpina1 | Alpha-1-antiproteinase | -0.67 | |
|  | Padi2 | Protein-arginine deiminase type-2 | -0.67 | |
|  | Nfasc | Isoform 2 of Neurofascin | -0.67 | |
|  | Aldh3b1 | Aldehyde dehydrogenase family 3 member B1 | -0.66 | |
|  | Marcks | Myristoylated alanine-rich C-kinase substrate | -0.64 | |
|  | Ttr | Transthyretin | -0.64 | |
|  | Gsn | Gelsolin | -0.63 | |
|  | Alb | Serum albumin | -0.62 | |
|  | Tac1 | protachykinin-1 | -0.62 | |
|  | Sirt2 | NAD-dependent protein deacetylase sirtuin-2 | -0.62 | |
|  | LOC100134871; LOC689064; LOC103694857 | Hemoglobin subunit beta-2 | -0.61 | |
|  | Ptp4a1; LOC100365697; LOC102547074; LOC103693189 | Protein tyrosine phosphatase type IVA 1 | -0.59 | |
|  | Tpm2 | Isoform 2 of Tropomyosin beta chain | -0.59 | |
|  | Jup | Junction plakoglobin | -0.57 | |
|  | Reep5 | Receptor expression-enhancing protein 5 | -0.56 | |
|  | Cd82 | CD82 antigen | -0.56 | |
|  | Serpina3c; Serpina3k | Serine protease inhibitor A3K | -0.56 | |
|  | Tf | Serotransferrin | -0.53 | |
|  | Gstp1 | Glutathione S-transferase P | -0.53 | |
|  | Map1a | Microtubule-associated protein 1A | -0.52 | |
|  | Gng7 | guanine nucleotide-binding protein G(I)/G(S)/G(O) subunit gamma-7 | -0.52 | |
|  | Qdpr | dihydropteridine reductase | -0.5 | |
|  | Aspa | aspartoacylase | -0.49 | |
|  | Ptma; LOC100359583 | Prothymosin alpha | -0.48 | |
|  | H2afz | Histone H2A.Z | -0.47 | |
|  | Ca2; Car2 | Carbonic anhydrase 2 | -0.47 | |
|  | Lap3 | cytosol aminopeptidase | -0.47 | |
|  | Wipf3 | WAS/WASL-interacting protein family member 3 | -0.47 | |
|  | Enpp6 | Ectonucleotide pyrophosphatase/phosphodiesterase family member 6 | -0.45 | |
|  | Sepw1; Selenow | selenoprotein w | -0.45 | |
|  | Gsta3; Gsta1; LOC102550391; LOC108348061 | glutathione S-transferase alpha-3 | -0.44 | |
|  | Slc4a1 | Band 3 anion transport protein | -0.44 | |
|  | Tbca | Tubulin-specific chaperone A | -0.42 | |
|  | Slc6a11 | Sodium- and chloride-dependent GABA transporter 3 | -0.42 | |
|  | Hba-a2; Hba1; Hba2 | Hemoglobin subunit alpha-1/2 | -0.42 | |
|  | Fah | Fumarylacetoacetase | -0.41 | |
|  | Eml1 | Echinoderm microtubule-associated protein-like 1 | -0.4 | |
|  | Atp5j | ATP synthase-coupling factor 6, mitochondrial | -0.4 | |
|  | Rer1 | Protein RER1 | -0.4 | |
|  | Tpm3 | Isoform 2 of Tropomyosin alpha-3 chain | -0.39 | |
|  | Atp5i | ATP synthase subunit e, mitochondrial | -0.39 | |
|  | Gpd1 | Glycerol-3-phosphate dehydrogenase [NAD(+)], cytoplasmic | -0.37 | |
|  | Hbb | Hemoglobin subunit beta-1 | -0.34 | |
|  | Gap43 | Neuromodulin | -0.34 | |
|  | App | Amyloid-beta A4 protein | -0.34 | |
|  | Rtn4 | Reticulon-4 | -0.33 | |
|  | Ddah2 | N(G),N(G)-dimethylarginine dimethylaminohydrolase 2 | -0.33 | |
| Upregulated | Phb2 | Prohibitin-2 | 0.33 |  |
|  | Slc8a1 | Isoform 2 of Sodium/calcium exchanger 1 | 0.33 |  |
|  | Anp32a | Acidic leucine-rich nuclear phosphoprotein 32 family member A | 0.33 |  |
|  | Dlgap3 | Disks large-associated protein 3 | 0.33 |  |
|  | Ncald | Neurocalcin-delta | 0.33 |  |
|  | COX2 | Cytochrome c oxidase subunit 2 | 0.34 |  |
|  | Pi4ka | phosphatidylinositol 4-kinase alpha | 0.34 |  |
|  | Atp2b4 | Isoform XA of Plasma membrane calcium-transporting ATPase 4 | 0.34 |  |
|  | Sh3gl3 | Endophilin-A3 | 0.34 |  |
|  | Pgam5 | Serine/threonine-protein phosphatase Pgam5, mitochondrial | 0.35 |  |
|  | Prkaca | cAMP-dependent protein kinase catalytic subunit alpha | 0.35 |  |
|  | Gls | Glutaminase kidney isoform, mitochondrial | 0.35 |  |
|  | Acot7 | Isoform 1 of Cytosolic acyl coenzyme A thioester hydrolase | 0.35 |  |
|  | Eef1a2 | Elongation factor 1-alpha 2 | 0.35 |  |
|  | Ilf3 | Interleukin enhancer-binding factor 3 | 0.35 |  |
|  | Slc1a3 | Excitatory amino acid transporter 1 | 0.35 |  |
|  | Ptprs | Receptor-type tyrosine-protein phosphatase S | 0.35 |  |
|  | Dld | Dihydrolipoyl dehydrogenase, mitochondrial | 0.36 |  |
|  | Vsnl1 | Visinin-like protein 1 | 0.36 |  |
|  | Camk2a | Calcium/calmodulin-dependent protein kinase type II subunit alpha | 0.36 |  |
|  | Sae1 | SUMO-activating enzyme subunit 1 | 0.36 |  |
|  | Pcyox1 | Prenylcysteine oxidase | 0.36 |  |
|  | Lrpprc | Leucine-rich PPR motif-containing protein, mitochondrial | 0.36 |  |
|  | Rpl11 | 60S ribosomal protein L11 | 0.37 |  |
|  | Matr3 | Matrin-3 | 0.37 |  |
|  | Pura | Transcriptional activator protein Pur-alpha | 0.37 |  |
|  | Rab3b | Ras-related protein Rab-3B | 0.37 |  |
|  | Cs | citrate synthase, mitochondrial | 0.38 |  |
|  | Eif4a3 | Eukaryotic initiation factor 4A-III | 0.38 |  |
|  | Hspa13 | Heat shock 70 kDa protein 13 | 0.38 |  |
|  | Plxna3 | Plexin-A3 | 0.38 |  |
|  | Ocrl | Inositol polyphosphate 5-phosphatase OCRL-1 | 0.38 |  |
|  | Gnaz | guanine nucleotide-binding protein G(z) subunit alpha | 0.39 |  |
|  | Arpc2 | Actin-related protein 2/3 complex subunit 2 | 0.39 |  |
|  | Rab2a | Ras-related protein Rab-2A | 0.39 |  |
|  | LOC100362640; Rps4x; Rps4x-ps9 | 40S ribosomal protein S4, X isoform | 0.39 |  |
|  | Poglut1 | Protein O-glucosyltransferase 1 | 0.39 |  |
|  | Sh3gl2 | Endophilin-A1 | 0.4 |  |
|  | Entpd2 | ectonucleoside triphosphate diphosphohydrolase 2 | 0.4 |  |
|  | Gapdh; LOC685186; LOC108351137 | glyceraldehyde-3-phosphate dehydrogenase | 0.41 |  |
|  | Dclk1 | serine/threonine-protein kinase DCLK1 | 0.41 |  |
|  | Rpl14 | 60S ribosomal protein L14 | 0.41 |  |
|  | Rbm3 | RNA-binding protein 3 | 0.41 |  |
|  | Ap1b1 | AP-1 complex subunit beta-1 | 0.42 |  |
|  | Ptbp2 | Polypyrimidine tract-binding protein 2 | 0.42 |  |
|  | Sec14l2 | SEC14-like protein 2 | 0.43 |  |
|  | Adrbk1; Grk2 | Beta-adrenergic receptor kinase 1 | 0.43 |  |
|  | Faah; LOC100911581 | Fatty-acid amide hydrolase 1 | 0.43 |  |
|  | LOC100360449; Rpl9; LOC100364457 | 60S ribosomal protein L9 | 0.44 |  |
|  | Prkar2b | cAMP-dependent protein kinase type II-beta regulatory subunit | 0.44 |  |
|  | Usp46 | Ubiquitin carboxyl-terminal hydrolase 46 | 0.44 |  |
|  | Rps9; LOC100909466; LOC103689992 | 40S ribosomal protein S9 | 0.45 |  |
|  | Maoa | Amine oxidase [flavin-containing] A | 0.45 |  |
|  | Cc2d1a | Coiled-coil and C2 domain-containing protein 1A | 0.45 |  |
|  | Rplp1; LOC100360522 | 60S acidic ribosomal protein P1 | 0.45 |  |
|  | Cnrip1 | CB1 cannabinoid receptor-interacting protein 1 | 0.45 |  |
|  | Hpcal4 | Hippocalcin-like protein 4 | 0.46 |  |
|  | Eif4a2 | eukaryotic initiation factor 4A-II | 0.46 |  |
|  | Ncs1 | neuronal calcium sensor 1 | 0.47 |  |
|  | Elavl2 | ELAV-like protein 2 | 0.47 |  |
|  | Vwa5a; LOC108348048 | von Willebrand factor A domain-containing protein 5A | 0.47 |  |
|  | Slc12a5 | Solute carrier family 12 member 5 | 0.48 |  |
|  | Mpp2 | MAGUK p55 subfamily member 2 | 0.48 |  |
|  | Opcml | Opioid-binding protein/cell adhesion molecule | 0.48 |  |
|  | Grm8 | Metabotropic glutamate receptor 8 | 0.48 |  |
|  | Syn2 | Synapsin-2 | 0.49 |  |
|  | Grin2b | Glutamate receptor ionotropic, NMDA 2B | 0.5 |  |
|  | Necab1 | N-terminal EF-hand calcium-binding protein 1 | 0.5 |  |
|  | Slc4a10 | Sodium-driven chloride bicarbonate exchanger | 0.5 |  |
|  | Nptxr | neuronal pentraxin receptor | 0.51 |  |
|  | Cpne7 | Copine-7 | 0.51 |  |
|  | LOC100362027; Rpl30 | 60S ribosomal protein L30 | 0.52 |  |
|  | Hnrnpa3 | Heterogeneous nuclear ribonucleoprotein A3 | 0.52 |  |
|  | Tomm34 | Mitochondrial import receptor subunit TOM34 | 0.52 |  |
|  | Prkra | Interferon-inducible double-stranded RNA-dependent protein kinase activator A | 0.52 |  |
|  | LOC687090; Tprg1l | Tumor protein p63-regulated gene 1-like protein | 0.54 |  |
|  | Hapln1 | Hyaluronan and proteoglycan link protein 1 | 0.54 |  |
|  | Hnrnpa1 | Heterogeneous nuclear ribonucleoprotein A1 | 0.55 |  |
|  | Timp2 | Metalloproteinase inhibitor 2 | 0.55 |  |
|  | Gaa | lysosomal alpha-glucosidase | 0.56 |  |
|  | Sfxn1 | Sideroflexin-1 | 0.56 |  |
|  | Gabra2 | Gamma-aminobutyric acid receptor subunit alpha-2 | 0.56 |  |
|  | Prkacb | cAMP-dependent protein kinase catalytic subunit beta | 0.57 |  |
|  | Ilf2 | Interleukin enhancer-binding factor 2 | 0.58 |  |
|  | ND2 | NADH-ubiquinone oxidoreductase chain 2 | 0.58 |  |
|  | Prepl | prolyl endopeptidase-like | 0.59 |  |
|  | Syngr1 | Synaptogyrin-1 | 0.59 |  |
|  | Dcps | M7GpppX diphosphatase | 0.59 |  |
|  | Grin1 | Glutamate receptor ionotropic, NMDA 1 | 0.6 |  |
|  | Atp4a | Potassium-transporting ATPase alpha chain 1 | 0.61 |  |
|  | Prkar2a | cAMP-dependent protein kinase type II-alpha regulatory subunit | 0.63 |  |
|  | Sub1 | Activated RNA polymerase II transcriptional coactivator p15 | 0.63 |  |
|  | Rab3c | ras-related protein Rab-3C | 0.64 |  |
|  | Gabrg3 | Gamma-aminobutyric acid receptor subunit gamma-3 | 0.64 |  |
|  | Cd59 | CD59 glycoprotein | 0.65 |  |
|  | Rab3a | Ras-related protein Rab-3A | 0.66 |  |
|  | Ddx21 | Nucleolar RNA helicase 2 | 0.68 |  |
|  | Gria2 | Isoform Flip of Glutamate receptor 2 | 0.69 |  |
|  |  | Isoform 5 of Ras/Rap GTPase-activating protein SynGAP | 0.71 |  |
|  | Ssbp3 | Single-stranded DNA-binding protein 3 | 0.71 |  |
|  | Aktip | AKT-interacting protein | 0.74 |  |
|  | Sv2b | Synaptic vesicle glycoprotein 2B | 0.75 |  |
|  | Hacl1 | 2-hydroxyacyl-CoA lyase 1 | 0.75 |  |
|  | Ctsz | Cathepsin Z | 0.79 |  |
|  | Ss18l1 | Calcium-responsive transcription coactivator | 0.79 |  |
|  | Gria1 | Glutamate receptor 1 | 0.87 |  |
|  | Slc17a7 | Vesicular glutamate transporter 1 | 0.87 |  |
|  | Arf3 | ADP-ribosylation factor 3 | 1 |  |
|  | Kcnj3 | G protein-activated inward rectifier potassium channel 1 | 1.03 |  |
|  | Mapk14 | Mitogen-activated protein kinase 14 | 1.12 |  |
|  | Cacng2 | Voltage-dependent calcium channel gamma-2 subunit | 1.24 |  |
|  | Ncbp1 | Nuclear cap-binding protein subunit 1 | 1.39 |  |
|  | Nras | GTPase NRas | 1.41 |  |
|  | Unc5a | Netrin receptor UNC5A | 1.47 |  |
|  | Ppapdc3; Plpp7 | Inactive phospholipid phosphatase 7 | 1.51 |  |
|  | Magmas-ps1 | Mitochondrial import inner membrane translocase subunit TIM16 | 1.84 |  |
|  | Crcp | DNA-directed RNA polymerase III subunit RPC9 | 1.97 |  |
|  | Arhgef1 | Rho guanine nucleotide exchange factor 1 | 2.04 |  |
|  | Timmdc1 | Complex I assembly factor TIMMDC1, mitochondrial | 2.05 |  |
|  | Sphkap | A-kinase anchor protein SPHKAP | 3.83 |  |

***Supplemental Table 4*. A comprehensive list of all proteins significantly downregulated or upregulated by mifepristone in the context of CIEV in male Wistar rats.** The gene symbols, protein names, and Log2FC are included in the table. Only those with a *P* ≤ 0.05 and a FC ≥ 1.25/1 or FC ≤ 1/1.25 are listed.

| CIEV Effects (CIEV-Placebo/Air-Placebo) | | | | | | | | |
| --- | --- | --- | --- | --- | --- | --- | --- | --- |
| Ingenuity  Canonical Pathways | **-log(*p*-value)** | **Ratio** | | **Activation z-score** | | **Molecules** | | |
| EIF2 Signaling | 10.4 | 0.0966 | | -3.162 | | EIF3A, EIF3H, EIF4A2, EIF4A3, ELP6, HNRNPA1, NRAS, PIK3R2, PPP1CB, RPL11, RPL14, RPL15, RPL18A, RPL26, RPL30, RPL9, RPS2, RPS4Y1, RPS9, RPSA | | |
| Synaptic Long Term Potentiation | 9.24 | 0.12 | | -2.84 | | CAMK2B, CREBBP, GNA11, GRIA1, GRIA2, GRIN1, GRIN2B, GRM2, GRM3, NRAS, PPP1CB, PPP1R14A, PRKACB, PRKAR2A, PRKAR2B | | |
| Eukaryotic Translation Initiation | 9.05 | 0.128 | | -3.742 | | EIF3A, EIF3H, EIF4A2, RPL11, RPL14, RPL15, RPL18A, RPL26, RPL30, RPL9, RPS2, RPS4Y1, RPS9, RPSA | | |
| Synaptogenesis Signaling Pathway | 9.02 | 0.0731 | | -3.838 | | AP2B1, ARPC2, CAMK2B, CREBBP, DLG4, GRIA1, GRIA2, GRIN1, GRIN2B, GRM2, GRM3, KALRN, LIMK1, MAPK14, NLGN1, NRAS, PIK3R2, PRKACB, PRKAR2A, PRKAR2B, RAC1, SYN2 | | |
| Eukaryotic Translation Elongation | 8.44 | 0.145 | | -3.464 | | EEF1A2, RPL11, RPL14, RPL15, RPL18A, RPL26, RPL30, RPL9, RPS2, RPS4Y1, RPS9, RPSA | | |
| Nonsense-Mediated Decay (NMD) | 8.25 | 0.124 | | -3.606 | | EIF4A3, NCBP1, RPL11, RPL14, RPL15, RPL18A, RPL26, RPL30, RPL9, RPS2, RPS4Y1, RPS9, RPSA | | |
| Neuropathic Pain Signaling in Dorsal Horn Neurons | 7.6 | 0.122 | | -2.309 | | CAMK2B, GRIA1, GRIA2, GRIN1, GRIN2B, GRM2, GRM3, PIK3R2, PRKACB, PRKAR2A, PRKAR2B, TAC1 | | |
| Glutamate Receptor Signaling | 7.53 | 0.159 | | -2.449 | | DLG4, GNG7, GRIA1, GRIA2, GRIN1, GRIN2B, GRM2, GRM3, SLC17A7, SLC1A3 | | |
| Eukaryotic Translation Termination | 7.43 | 0.134 | | -3.317 | | RPL11, RPL14, RPL15, RPL18A, RPL26, RPL30, RPL9, RPS2, RPS4Y1, RPS9, RPSA | | |
| SRP-dependent cotranslational protein targeting to membrane | 7.4 | 0.118 | | -3.464 | | RPL11, RPL14, RPL15, RPL18A, RPL26, RPL30, RPL9, RPN2, RPS2, RPS4Y1, RPS9, RPSA | | |
| Glutaminergic Receptor Signaling Pathway (Enhanced) | 7.36 | 0.0647 | | -2.683 | | CACNG2, CAMK2B, CREBBP, DGKB, GABRA2, GABRG3, GRIA1, GRIA2, GRIN1, GRIN2B, GRM2, GRM3, MAPK14, PIK3R2, PRKACB, PRKAR2A, PRKAR2B, SCN4B, SHANK3, SLC1A3 | | |
| Response of EIF2AK4 (GCN2) to amino acid deficiency | 6.96 | 0.121 | | -3.317 | | RPL11, RPL14, RPL15, RPL18A, RPL26, RPL30, RPL9, RPS2, RPS4Y1, RPS9, RPSA | | |
| Neurovascular Coupling Signaling Pathway | 6.87 | 0.0751 | | -3.5 | | CACNG2, CHRM1, CHRM4, GABRA2, GABRG3, GRIA1, GRIA2, GRIN1, GRIN2B, KCNJ3, NOS1, PPP1CB, PRKACB, PRKAR2A, PRKAR2B, SLC1A3 | | |
| Selenoamino acid metabolism | 6.86 | 0.118 | | -3.317 | | RPL11, RPL14, RPL15, RPL18A, RPL26, RPL30, RPL9, RPS2, RPS4Y1, RPS9, RPSA | | |
| Synaptic adhesion-like molecules | 6.33 | 0.286 | | -2.449 | | DLG4, FLOT2, GRIA1, GRIN1, GRIN2B, PTPRS | | |
| Dopamine-DARPP32 Feedback in cAMP Signaling | 5.57 | 0.0734 | | -2.111 | | CACNG2, CREBBP, GRIN1, GRIN2B, KCNJ3, NOS1, PPP1CB, PPP1R14A, PPP1R1B, PPP2CB, PRKACB, PRKAR2A, PRKAR2B | | |
| Calcium Signaling | 4.92 | 0.064 | | -3.051 | | ATP2B4, CACNG2, CAMK2B, CREBBP, GRIA1, GRIA2, GRIN1, GRIN2B, PNCK, PRKACB, PRKAR2A, PRKAR2B, SLC8A1 | | |
| AMPK Signaling | 4.88 | 0.0593 | | -2.53 | | AK1, CREBBP, ELAVL1, GNA11, GNAZ, GNG7, MAPK14, PFKL, PIK3R2, PPP2CB, PRKACB, PRKAR2A, PRKAR2B, RAB27A | | |
| Neuroinflammation Signaling Pathway | 4.68 | 0.0538 | | -3.162 | | B2M, CALB2, CREBBP, GABRA2, GABRG3, GRIA1, GRIN1, GRIN2B, KCNJ3, MAPK14, PIK3R2, RAC1, SLC1A3, SLC6A11, VCAM1 | | |
| Major pathway of rRNA processing in the nucleolus and cytosol | 4.29 | 0.0647 | | -3.317 | | RPL11, RPL14, RPL15, RPL18A, RPL26, RPL30, RPL9, RPS2, RPS4Y1, RPS9, RPSA | | |
| EPH-Ephrin signaling | 4.29 | 0.0909 | | -2.121 | | AP2B1, ARPC2, CLTC, GRIN1, GRIN2B, KALRN, LIMK1, RAC1 | | |
| IL-1 Signaling | 4.05 | 0.0842 | | -2.236 | | ECSIT, GNA11, GNAZ, GNG7, MAPK14, PRKACB, PRKAR2A, PRKAR2B | | |
| G Beta Gamma Signaling | 3.96 | 0.072 | | -2.333 | | CACNG2, GNA11, GNAZ, GNG7, KCNJ3, NRAS, PRKACB, PRKAR2A, PRKAR2B | | |
| Neurexins and neuroligins | 3.92 | 0.115 | | -2.449 | | DLG4, DLGAP3, GRIN1, GRIN2B, NLGN1, SHANK3 | | |
| CREB Signaling in Neurons | 3.9 | 0.0371 | | -2.828 | | ADGRG1, ADGRL3, CACNG2, CAMK2B, CHRM1, CHRM4, CREBBP, GNA11, GNAZ, GNG7, GRIA1, GRIA2, GRIN1, GRIN2B, GRM2, GRM3, NRAS, PIK3R2, PRKACB, PRKAR2A, PRKAR2B | | |
| G-Protein Coupled Receptor Signaling | 3.81 | 0.0348 | | -2.711 | | ADGRG1, ADGRL3, CAMK2B, CHRM1, CHRM4, CREBBP, ENPP6, GNA11, GNAZ, GNG7, GRK2, GRM2, GRM3, HCN3, MAPK14, NRAS, PDE10A, PIK3R2, PRKACB, PRKAR2A, PRKAR2B, PTK2B, RAC1 | | |
| Sertoli Cell-Germ Cell Junction Signaling Pathway (Enhanced) | 3.8 | 0.0531 | | -2.887 | | ARPC2, CAMK2B, CLDN11, JUP, MAPK14, NRAS, PIK3R2, PPP2CB, PRKACB, PRKAR2A, PRKAR2B, RAC1 | | |
| Ephrin Receptor Signaling | 3.68 | 0.0553 | | -2.111 | | ARPC2, CREBBP, GNA11, GNAZ, GNG7, GRIN1, GRIN2B, KALRN, LIMK1, NRAS, RAC1 | | |
| ERK/MAPK Signaling | 3.66 | 0.055 | | -2.111 | | CREBBP, NRAS, PIK3R2, PPP1CB, PPP1R14A, PPP2CB, PRKACB, PRKAR2A, PRKAR2B, PTK2B, RAC1 | | |
| Cardiac Hypertrophy Signaling | 3.45 | 0.0486 | | -2.121 | | CACNG2, CREBBP, GNA11, GNAZ, GNG7, MAPK14, NRAS, PIK3R2, PRKACB, PRKAR2A, PRKAR2B, RAC1 | | |
| Renin-Angiotensin Signaling | 3.38 | 0.0672 | | -2.121 | | MAPK14, NRAS, PIK3R2, PRKACB, PRKAR2A, PRKAR2B, PTK2B, RAC1 | | |
| Acetylcholine Receptor Signaling Pathway | 3.36 | 0.0546 | | -2.53 | | CACNG2, CAMK2B, CHRM1, CHRM4, CREBBP, GNA11, PIK3R2, PRKACB, PRKAR2A, PRKAR2B | | |
| Synaptic Long Term Depression | 3.34 | 0.0543 | | -3.162 | | CACNG2, GNA11, GNAZ, GRIA1, GRIA2, GRM2, GRM3, NOS1, NRAS, PPP2CB | | |
| Insulin Secretion Signaling Pathway | 3.27 | 0.0463 | | -2.53 | | CACNG2, CAMK2B, CREBBP, EIF4A2, EIF4A3, ELP6, GNA11, MAPK14, PIK3R2, PRKACB, PRKAR2A, PRKAR2B | | |
| Sleep REM Signaling Pathway | 3.1 | 0.0693 | | -2.646 | | CHRM1, CHRM4, CREBBP, LIMK1, PRKACB, PRKAR2A, PRKAR2B | | |
| Estrogen Receptor Signaling | 3.09 | 0.0385 | | -3.051 | | CACNG2, CREBBP, DLG4, GNA11, GNAZ, GNG7, HNRNPD, LIMK1, MT-ND2, NRAS, PIK3R2, PPP1CB, PRKACB, PRKAR2A, PRKAR2B | | |
| Cargo concentration in the ER | 2.85 | 0.121 | | -2 | | CD59, CTSZ, GRIA1, TMED2 | | |
| Signaling by Rho Family GTPases | 2.78 | 0.043 | | -2.121 | | ARHGEF1, ARPC2, EZR, GNA11, GNAZ, GNG7, LIMK1, PI4KA, PIK3R2, PTK2B, RAC1 | | |
| BMP signaling pathway | 2.72 | 0.069 | | -2.449 | | CREBBP, MAPK14, NRAS, PRKACB, PRKAR2A, PRKAR2B | | |
| Adrenomedullin signaling pathway | 2.66 | 0.0476 | | -3 | | CRCP, GNA11, MAPK14, NRAS, PIK3R2, PRKACB, PRKAR2A, PRKAR2B, PTK2B | | |
| Androgen Signaling | 2.65 | 0.0519 | | -2.236 | | CACNG2, CREBBP, GNA11, GNAZ, GNG7, PRKACB, PRKAR2A, PRKAR2B | | |
| Neurotransmitter release cycle | 2.58 | 0.103 | | -2 | | MAOA, SLC17A7, SLC1A3, SYN2 | | |
| Melanocyte Development and Pigmentation Signaling | 2.52 | 0.0632 | | -2.449 | | CREBBP, NRAS, PIK3R2, PRKACB, PRKAR2A, PRKAR2B | | |
| Regulation of mRNA stability by proteins that bind AU-rich elements | 2.5 | 0.0976 | | -2 | | ELAVL1, HNRNPD, MAPK14, SET | | |
| trans-Golgi Network Vesicle Budding | 2.41 | 0.0714 | | -2.236 | | AP1B1, CLTC, CTSZ, OCRL, SH3GL2 | | |
| nNOS Signaling in Neurons | 2.38 | 0.0909 | | -2 | | DLG4, GRIN1, GRIN2B, NOS1 | | |
| RHO GTPase cycle | 2.33 | 0.0331 | | -2.138 | | ACTN1, ARHGEF1, CLTC, DDX39B, FLOT2, JUP, KALRN, NISCH, OCRL, PIK3R2, PTK2B, RAC1, TXNL1, WIPF3 | | |
| PFKFB4 Signaling Pathway | 2.31 | 0.087 | | -2 | | CREBBP, PRKACB, PRKAR2A, PRKAR2B | | |
| MYC Mediated Apoptosis Signaling | 2.28 | 0.0851 | | -2 | | CREBBP, PRKACB, PRKAR2A, PRKAR2B | | |
| Keratinization | 2.25 | 0.0556 | | 2.449 | | JUP, KRT1, KRT17, KRT5, KRT72, KRT8 | | |
| Nitric Oxide Signaling in the Cardiovascular System | 2.17 | 0.0536 | | -2.236 | | CACNG2, CHRM1, PIK3R2, PRKACB, PRKAR2A, PRKAR2B | | |
| Eicosanoid Signaling | 2.14 | 0.0373 | | -2.53 | | AP1B1, CREBBP, GNA11, GNG7, MAPK14, NRAS, PIK3R2, PRKACB, PRKAR2A, PRKAR2B | | |
| Cytoprotection by HMOX1 | 1.96 | 0.069 | | -2 | | CREBBP, LRPPRC, MT-CO2, TACO1 | | |
| UFMylation Signaling Pathway | 1.93 | 0.0678 | | -2 | | EIF4A2, EIF4A3, ELP6, RPL26 | | |
| IL-33 Signaling Pathway | 1.88 | 0.0417 | | -2.646 | | CREBBP, MAPK14, PIK3R2, PRKACB, PRKAR2A, PRKAR2B, VCAM1 | | |
| Protein Sorting Signaling Pathway | 1.86 | 0.0414 | | -2.646 | | AP1B1, CLTC, COPB1, PRKACB, PRKAR2A, PRKAR2B, TMED2 | | |
| VEGF Signaling | 1.86 | 0.0526 | | -2 | | ACTN1, ELAVL1, NRAS, PIK3R2, PTK2B | | |
| Processing of Capped Intron-Containing Pre-mRNA | 1.85 | 0.0356 | | -2.333 | | DDX39B, EIF4A3, HNRNPA1, HNRNPA2B1, HNRNPD, NCBP1, PUF60, SDE2, SNRPB | | |
| S100 Family Signaling Pathway | 1.81 | 0.0262 | | -3.771 | | ADGRG1, ADGRL3, CACNG2, CAMK2B, CHRM1, CHRM4, CREBBP, EZR, GNA11, GRM2, GRM3, MAPK14, PIK3R2, PRKACB, PRKAR2A, PRKAR2B, RAC1, VCAM1 | | |
| White Adipose Tissue Browning Pathway | 1.81 | 0.0448 | | -2.449 | | CACNG2, CREBBP, MAPK14, PRKACB, PRKAR2A, PRKAR2B | | |
| Transport of inorganic cations/anions and amino acids/oligopeptides | 1.77 | 0.05 | | -2.236 | | SLC12A5, SLC17A7, SLC1A3, SLC4A10, SLC8A1 | | |
| Apelin Endothelial Signaling Pathway | 1.77 | 0.0438 | | -2 | | GNA11, GNAZ, GNG7, NRAS, PIK3R2, VCAM1 | | |
| Melatonin Signaling | 1.77 | 0.0606 | | -2 | | CAMK2B, PRKACB, PRKAR2A, PRKAR2B | | |
| IGF-1 Signaling | 1.75 | 0.0495 | | -2.236 | | NRAS, PIK3R2, PRKACB, PRKAR2A, PRKAR2B | | |
| COPII-mediated vesicle transport | 1.68 | 0.0571 | | -2 | | CD59, CTSZ, GRIA1, TMED2 | | |
| FLT3 Signaling in Hematopoietic Progenitor Cells | 1.5 | 0.05 | | -2 | | CREBBP, MAPK14, NRAS, PIK3R2 | | |
| Clathrin-mediated endocytosis | 1.47 | 0.0417 | | -2.236 | | AP2B1, ARPC2, CLTC, OCRL, SH3GL2 | | |
| Molecular Mechanisms of Cancer | 1.46 | 0.0235 | | -2.828 | | ADGRG1, ADGRL3, ARHGEF1, CAMK2B, CHRM1, CHRM4, CREBBP, GNA11, GNAZ, GNG7, GRM2, GRM3, MAPK14, NRAS, PIK3R2, PRKACB, PRKAR2A, PRKAR2B, RAC1 | | |
| RNA Polymerase II Transcription | 1.37 | 0.0391 | | -2.236 | | DDX39B, EIF4A3, NCBP1, SNRPB, SSRP1 | | |
| Phagosome Formation | 1.31 | 0.024 | | -3.357 | | ADGRG1, ADGRL3, AP1B1, ARPC2, CHRM1, CHRM4, GRM2, GRM3, LIMK1, NRAS, OCRL, PI4KA, PIK3R2, PTK2B, RAC1 | | |
| CIEV Effects in the Context of Mifepristone (CIEV-Mifepristone/Air-Mifepristone) | | | | | | | | |
| Ingenuity  Canonical Pathways | **-log(*p*-value)** | | **Ratio** | | **Activation z-score** | | **Molecules** | |
| LXR/RXR Activation | 6.69 | | 0.0541 | | -2.449 | | AMBP,APOA1,C3,SERPINA1,TF,TTR | |
| DHCR24 Signaling Pathway | 6.34 | | 0.0472 | | -2.449 | | AMBP,APOA1,C3,SERPINA1,TF,TTR | |
| Post-translational protein phosphorylation | 4.04 | | 0.0392 | | -2 | | APOA1,C3,SERPINA1,TF | |
| Regulation of Insulin-like Growth Factor (IGF) transport and uptake by IGFBPs | 3.81 | | 0.0342 | | -2 | | APOA1,C3,SERPINA1,TF | |
| Response to elevated platelet cytosolic Ca2+ | 3.69 | | 0.0317 | | -2 | | APOA1,CD9,SERPINA1,TF | |
| Mifepristone Effects in the Context of CIEV (CIEV-Mifepristone/CIEV-Placebo) | | | | | | | | |
| Ingenuity Canonical Pathways | **-log(*p*-value)** | **Ratio** | | **Activation z-score** | | **Molecules** | |  |
| Activation of NMDA receptors and postsynaptic events | 10.1 | 0.158 | | 2.646 | | CAMK2A, CAMK2D, GRIA1, GRIA2, GRIN1, GRIN2B, NEFL, NRAS, PRKACA, PRKACB, PRKAR2A, PRKAR2B | |  |
| Neuropathic Pain Signaling in Dorsal Horn Neurons | 8.83 | 0.122 | | 2.309 | | CAMK2A, CAMK2D, GRIA1, GRIA2, GRIN1, GRIN2B, GRM8, PRKACA, PRKACB, PRKAR2A, PRKAR2B, TAC1 | |  |
| Synaptic Long Term Potentiation | 8.63 | 0.104 | | 3.051 | | CAMK2A, CAMK2D, GRIA1, GRIA2, GRIN1, GRIN2B, GRM8, NRAS, PPP1R14A, PRKACA, PRKACB, PRKAR2A, PRKAR2B | |  |
| Calcium Signaling | 7.81 | 0.0739 | | 3.051 | | ATP2B4, CACNG2, CAMK2A, CAMK2D, GRIA1, GRIA2, GRIN1, GRIN2B, PRKACA, PRKACB, PRKAR2A, PRKAR2B, SLC8A1, Tpm2, Tpm3 | |  |
| Glutaminergic Receptor Signaling Pathway (Enhanced) | 7.64 | 0.0583 | | 2.357 | | CACNG2, CAMK2A, CAMK2D, GABRA2, GABRG3, GLS, GRIA1, GRIA2, GRIN1, GRIN2B, GRM8, MAPK14, PRKACA, PRKACB, PRKAR2A, PRKAR2B, SCN4B, SLC1A3 | |  |
| Neurovascular Coupling Signaling Pathway | 7.53 | 0.0704 | | 2.84 | | APP, CACNG2, ENTPD2, GABRA2, GABRG3, GRIA1, GRIA2, GRIN1, GRIN2B, KCNJ3, PRKACA, PRKACB, PRKAR2A, PRKAR2B, SLC1A3 | |  |
| Glutamate Receptor Signaling | 7.35 | 0.143 | | 2.236 | | GLS, GNG7, GRIA1, GRIA2, GRIN1, GRIN2B, GRM8, SLC17A7, SLC1A3 | |  |
| Synaptogenesis Signaling Pathway | 7.05 | 0.0565 | | 3.638 | | ARPC2, CAMK2A, CAMK2D, GRIA1, GRIA2, GRIN1, GRIN2B, GRM8, MAPK14, NRAS, PRKACA, PRKACB, PRKAR2A, PRKAR2B, RAB3A, SYN2, SYNGAP1 | |  |
| Nonsense-Mediated Decay (NMD) | 5.43 | 0.0857 | | 3 | | EIF4A3, NCBP1, RPL11, RPL14, RPL30, RPL9, RPLP1, RPS4Y1, RPS9 | |  |
| Neurotransmitter release cycle | 5.27 | 0.154 | | 2.449 | | GLS, MAOA, RAB3A, SLC17A7, SLC1A3, SYN2 | |  |
| Eukaryotic Translation Elongation | 5.27 | 0.0964 | | 2.828 | | EEF1A2, RPL11, RPL14, RPL30, RPL9, RPLP1, RPS4Y1, RPS9 | |  |
| Dopamine-DARPP32 Feedback in cAMP Signaling | 5.14 | 0.0621 | | 2.333 | | CACNG2, GRIN1, GRIN2B, KCNJ3, PPP1R14A, PPP1R1B, PPP2CB, PRKACA, PRKACB, PRKAR2A, PRKAR2B | |  |
| G Beta Gamma Signaling | 4.81 | 0.072 | | 2.333 | | CACNG2, GNAZ, GNG7, KCNJ3, NRAS, PRKACA, PRKACB, PRKAR2A, PRKAR2B | |  |
| EIF2 Signaling | 4.5 | 0.0531 | | 2.449 | | EIF4A2, EIF4A3, HNRNPA1, NRAS, RPL11, RPL14, RPL30, RPL9, RPLP1, RPS4Y1, RPS9 | |  |
| Eukaryotic Translation Initiation | 4.39 | 0.0734 | | 2.828 | | EIF4A2, RPL11, RPL14, RPL30, RPL9, RPLP1, RPS4Y1, RPS9 | |  |
| Role of NFAT in Cardiac Hypertrophy | 4.34 | 0.0509 | | 2.111 | | CACNG2, CAMK2A, CAMK2D, GNG7, MAPK14, NRAS, PRKACA, PRKACB, PRKAR2A, PRKAR2B, SLC8A1 | |  |
| Eukaryotic Translation Termination | 4.33 | 0.0854 | | 2.646 | | RPL11, RPL14, RPL30, RPL9, RPLP1, RPS4Y1, RPS9 | |  |
| Synaptic adhesion-like molecules | 4.04 | 0.19 | | 2 | | GRIA1, GRIN1, GRIN2B, PTPRS | |  |
| Response of EIF2AK4 (GCN2) to amino acid deficiency | 4.04 | 0.0769 | | 2.646 | | RPL11, RPL14, RPL30, RPL9, RPLP1, RPS4Y1, RPS9 | |  |
| Selenoamino acid metabolism | 3.98 | 0.0753 | | 2.646 | | RPL11, RPL14, RPL30, RPL9, RPLP1, RPS4Y1, RPS9 | |  |
| IL-1 Signaling | 3.92 | 0.0737 | | 2.236 | | GNAZ, GNG7, MAPK14, PRKACA, PRKACB, PRKAR2A, PRKAR2B | |  |
| SRP-dependent cotranslational protein targeting to membrane | 3.73 | 0.0686 | | 2.646 | | RPL11, RPL14, RPL30, RPL9, RPLP1, RPS4Y1, RPS9 | |  |
| Insulin Secretion Signaling Pathway | 3.64 | 0.0425 | | 2.53 | | CACNG2, CAMK2A, CAMK2D, DLD, EIF4A2, EIF4A3, MAPK14, PRKACA, PRKACB, PRKAR2A, PRKAR2B | |  |
| Amyotrophic Lateral Sclerosis Signaling | 3.5 | 0.0631 | | 2 | | CACNG2, GRIA1, GRIA2, GRIN1, GRIN2B, NEFH, NEFL | |  |
| Sertoli Cell-Germ Cell Junction Signaling Pathway (Enhanced) | 3.49 | 0.0442 | | 3.162 | | ARPC2, CLDN11, JUP, MAPK14, NRAS, PPP2CB, PRKACA, PRKACB, PRKAR2A, PRKAR2B | |  |
| Gustation Pathway | 3.48 | 0.0484 | | 2.333 | | CACNG2, ENTPD2, GABRA2, GABRG3, PRKACA, PRKACB, PRKAR2A, PRKAR2B, SCN4B | |  |
| AMPK Signaling | 3.35 | 0.0424 | | 2.828 | | GNAZ, GNG7, MAPK14, PPP2CB, PRKACA, PRKACB, PRKAR2A, PRKAR2B, RAB2A, RAB3A | |  |
| BMP signaling pathway | 3.28 | 0.069 | | 2.449 | | MAPK14, NRAS, PRKACA, PRKACB, PRKAR2A, PRKAR2B | |  |
| Amyloid fiber formation | 3.15 | 0.082 | | -2.236 | | APOA1, APP, GSN, H2AZ1, TTR | |  |
| Major pathway of rRNA processing in the nucleolus and cytosol | 3.07 | 0.0471 | | 2.828 | | DDX21, RPL11, RPL14, RPL30, RPL9, RPLP1, RPS4Y1, RPS9 | |  |
| CREB Signaling in Neurons | 3.01 | 0.0283 | | 2.138 | | CACNG2, CAMK2A, CAMK2D, FGFR1, GNAZ, GNG7, GRIA1, GRIA2, GRIN1, GRIN2B, GRM8, NRAS, PRKACA, PRKACB, PRKAR2A, PRKAR2B | |  |
| Apelin Pancreas Signaling Pathway | 2.74 | 0.0889 | | -2 | | PRKACA, PRKACB, PRKAR2A, PRKAR2B | |  |
| PFKFB4 Signaling Pathway | 2.71 | 0.087 | | 2 | | PRKACA, PRKACB, PRKAR2A, PRKAR2B | |  |
| MYC Mediated Apoptosis Signaling | 2.67 | 0.0851 | | 2 | | PRKACA, PRKACB, PRKAR2A, PRKAR2B | |  |
| Androgen Signaling | 2.66 | 0.0455 | | 2.236 | | CACNG2, GNAZ, GNG7, PRKACA, PRKACB, PRKAR2A, PRKAR2B | |  |
| Renin-Angiotensin Signaling | 2.57 | 0.0504 | | 2.449 | | MAPK14, NRAS, PRKACA, PRKACB, PRKAR2A, PRKAR2B | |  |
| Phototransduction Pathway | 2.54 | 0.0784 | | 2 | | PRKACA, PRKACB, PRKAR2A, PRKAR2B | |  |
| Response to elevated platelet cytosolic Ca2+ | 2.45 | 0.0476 | | -2.449 | | ALB, APOA1, APP, CD9, SERPINA1, TF | |  |
| RAB geranylgeranylation | 2.31 | 0.0678 | | 2 | | RAB2A, RAB3A, RAB3B, RAB3C | |  |
| Melanocyte Development and Pigmentation Signaling | 2.3 | 0.0526 | | 2.236 | | NRAS, PRKACA, PRKACB, PRKAR2A, PRKAR2B | |  |
| Synaptic Long Term Depression | 2.24 | 0.038 | | 2.646 | | CACNG2, GNAZ, GRIA1, GRIA2, GRM8, NRAS, PPP2CB | |  |
| PXR/RXR Activation | 2.21 | 0.0635 | | 2 | | PRKACA, PRKACB, PRKAR2A, PRKAR2B | |  |
| IGF-1 Signaling | 2.19 | 0.0495 | | 2.236 | | NRAS, PRKACA, PRKACB, PRKAR2A, PRKAR2B | |  |
| Adrenomedullin signaling pathway | 2.18 | 0.037 | | 2.646 | | CRCP, MAPK14, NRAS, PRKACA, PRKACB, PRKAR2A, PRKAR2B | |  |
| Post-translational protein phosphorylation | 2.17 | 0.049 | | -2.236 | | ALB, APOA1, APP, SERPINA1, TF | |  |
| Keratinization | 2.07 | 0.0463 | | -2.236 | | JUP, KRT1, KRT17, KRT5, KRT8 | |  |
| GPCR-Mediated Integration of Enteroendocrine Signaling Exemplified by an L Cell | 2.05 | 0.0571 | | 2 | | PRKACA, PRKACB, PRKAR2A, PRKAR2B | |  |
| ERK/MAPK Signaling | 2.05 | 0.035 | | 2.646 | | NRAS, PPP1R14A, PPP2CB, PRKACA, PRKACB, PRKAR2A, PRKAR2B | |  |
| Inhibition of ARE-Mediated mRNA Degradation Pathway | 2.02 | 0.0387 | | 2.449 | | MAPK14, PPP2CB, PRKACA, PRKACB, PRKAR2A, PRKAR2B | |  |
| Nitric Oxide Signaling in the Cardiovascular System | 2 | 0.0446 | | 2 | | CACNG2, PRKACA, PRKACB, PRKAR2A, PRKAR2B | |  |
| Regulation of Insulin-like Growth Factor (IGF) transport and uptake by IGFBPs | 1.93 | 0.0427 | | -2.236 | | ALB, APOA1, APP, SERPINA1, TF | |  |
| Eicosanoid Signaling | 1.88 | 0.0299 | | 2.121 | | AP1B1, GNG7, MAPK14, NRAS, PRKACA, PRKACB, PRKAR2A, PRKAR2B | |  |
| IL-33 Signaling Pathway | 1.86 | 0.0357 | | 2.236 | | H2AZ1, MAPK14, PRKACA, PRKACB, PRKAR2A, PRKAR2B | |  |
| ABRA Signaling Pathway | 1.82 | 0.0488 | | -2 | | GSN, PPP1R14A, Tpm2, Tpm3 | |  |
| Ephrin A Signaling | 1.71 | 0.0376 | | -2.236 | | APP, PRKACA, PRKACB, PRKAR2A, PRKAR2B | |  |
| Cell junction organization | 1.71 | 0.0455 | | -2 | | CD151, CLDN11, JUP, KRT5 | |  |
| Sperm Motility | 1.64 | 0.029 | | 2 | | FGFR1, GNAZ, GNG7, PRKACA, PRKACB, PRKAR2A, PRKAR2B | |  |
| Adrenergic Receptor Signaling Pathway (Enhanced) | 1.62 | 0.0316 | | 2.449 | | CACNG2, MAOA, PRKACA, PRKACB, PRKAR2A, PRKAR2B | |  |
| Sleep REM Signaling Pathway | 1.52 | 0.0396 | | 2 | | PRKACA, PRKACB, PRKAR2A, PRKAR2B | |  |
| Hedgehog 'off' state | 1.47 | 0.0381 | | 2 | | PRKACA, PRKACB, PRKAR2A, PRKAR2B | |  |
| Ovarian Cancer Signaling | 1.47 | 0.0325 | | 2.236 | | NRAS, PRKACA, PRKACB, PRKAR2A, PRKAR2B | |  |

***Supplemental Table 5*. Ingenuity canonical pathways significantly altered by CIEV, CIEV in the context of mifepristone, and mifepristone in the context of CIEV in male Wistar rats.** The table includes all the Ingenuity canonical pathways altered, -log(*p*-value), ratio, activation z-score, and the proteins involved in the respective pathway. Only those with a *p-*value ≤ 0.05 and |activation z-score| ≥ 2 are considered as significant changes and included in this table. Mifepristone did not significantly alter any canonical pathways.


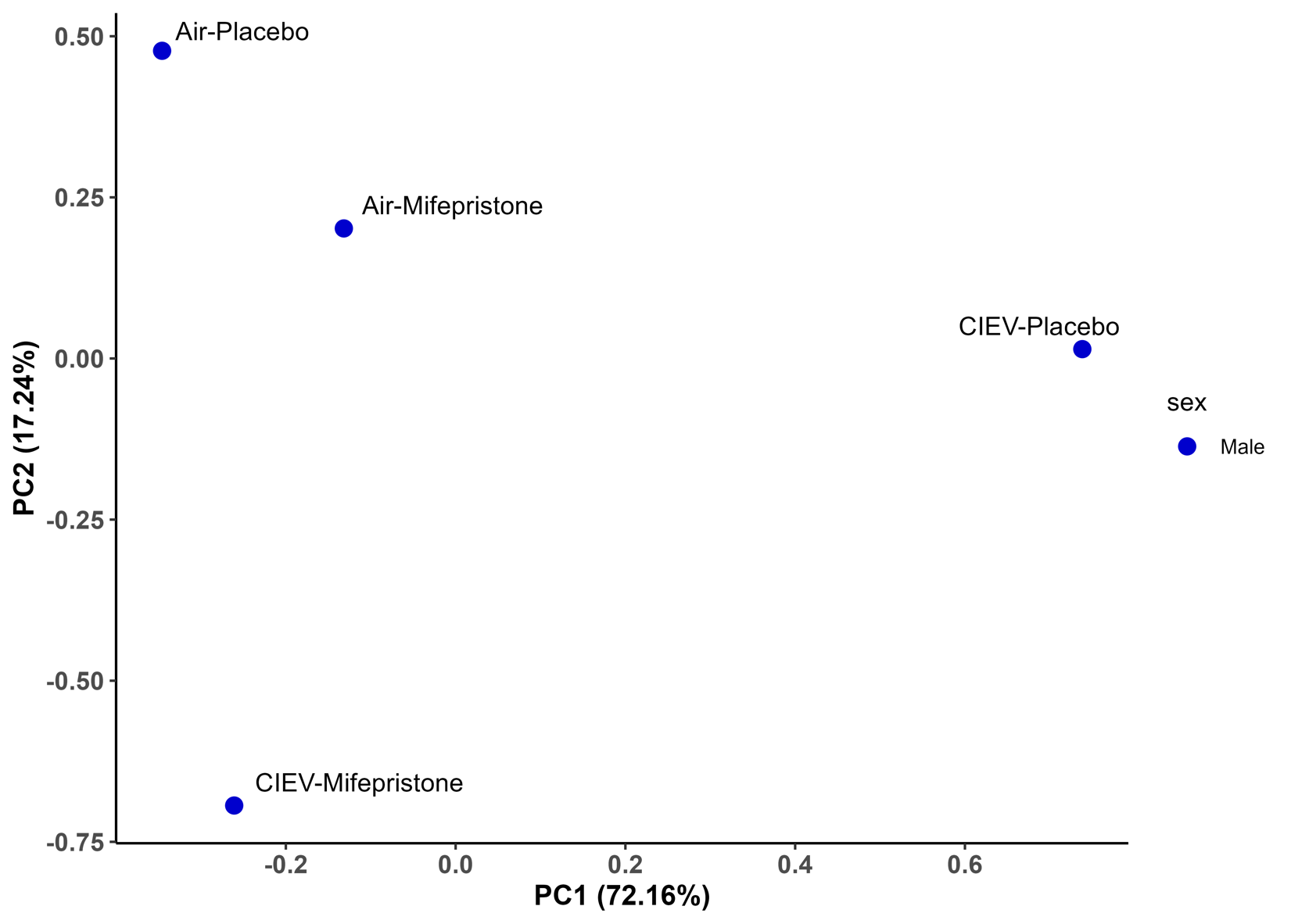


***Supplemental Figure 1.* Principal Component Analysis (PCA) score plot of proteomics data of male Wistar rats.** The PCA plot represents >3,000 proteins that were expressed in all groups. Proteomic profiles of CIEV and air animals separate along principal component 1, which explains 72.16% of variability in our data set. Proteomic profiles of mifepristone and placebo animals separate along principal component 2, which explains 17.24% of variability in our data set. Air-Placebo (*n* = 6); Air-Mifepristone (*n* = 6); CIEV-Placebo (*n =* 4); CIEV-Mifepristone (*n* = 4).

| CIEV-Placebo vs. Air-Placebo | | | |
| --- | --- | --- | --- |
|  | **Gene Symbol** | **Protein Name** | **Log2FC** |
| Downregulated | Cd38 | ADP-ribosyl cyclase/cyclic ADP-ribose hydrolase 1 | -0.78 |
|  | Pgam2 | ADP-ribosyl cyclase/cyclic ADP-ribose hydrolase 1 | -0.73 |
|  | GLTP | Glycolipid transfer protein | -0.64 |
|  | Fam98a | Protein FAM98A | -0.62 |
|  | Plp1 | Myelin proteolipid protein | -0.58 |
|  | Rap1a | Ras-related protein Rap-1A | -0.57 |
|  | Alb | Albumin | -0.56 |
|  | Myo1d | Unconventional myosin-Id | -0.55 |
|  | Tph2 | Tryptophan 5-hydroxylase 2 | -0.53 |
|  | Cd9 | CD9 antigen | -0.52 |
|  | Aimp2 | Aminoacyl tRNA synthase complex-interacting multifunctional protein 2 | -0.50 |
|  | Sirt2 | NAD-dependent protein deacetylase sirtuin-2 | -0.47 |
|  | Rap1b | Ras-related protein Rap-1b | -0.46 |
|  | Enpp6 | Glycerophosphocholine cholinephosphodiesterase ENPP6 | -0.46 |
|  | Gpd1 | Glycerol-3-phosphate dehydrogenase [NAD(+)], cytoplasmic | -0.44 |
|  | Eif2b4 | Translation initiation factor eIF-2B subunit delta | -0.43 |
|  | Padi2 | Protein-arginine deiminase type-2 | -0.40 |
|  | Anxa6 | Annexin A6 | -0.38 |
|  | Lgals1 | Galectin-1 | -0.35 |
| Upregulated | Snca | Alpha-synuclein | 0.34 |
|  | Scg2 | Secretogranin-2 | 0.36 |
|  | Cpe | Carboxypeptidase | 0.37 |
|  | Ptk2b | Protein-tyrosine kinase 2-beta | 0.39 |
|  | Calm3 | Calmodulin-3 | 0.40 |
|  | Clu | Clusterin | 0.42 |
|  | Basp1 | Brain acid soluble protein 1 | 0.44 |
|  | Sst | Somatostatin | 0.46 |
|  | Camk2d | Calcium/calmodulin-dependent protein kinase type II subunit delta | 0.52 |
|  | Tesk1 | Dual specificity testis-specific protein kinase 1 | 0.52 |
|  | Nucb2 | Nucleobindin-2 | 0.55 |
|  | Nucks1 | Nuclear ubiquitous casein and cyclin-dependent kinase substrate 1 | 0.58 |
|  | Penk | Proenkephalin-A | 0.74 |
|  | Ptms | Parathymosin | 0.81 |
|  | Ptma | Prothymosin alpha | 0.88 |
|  | Nptxr | Neuronal pentraxin receptor | 0.90 |
|  | Vgf | Neurosecretory protein VGF | 0.96 |
|  | Cck | Cholecystokinin | 0.98 |

***Supplemental Table 6*. A comprehensive list of all proteins significantly downregulated or upregulated by CIEV in female Wistar rats.** The gene symbols, protein names, and Log2FC are included in the table. Only those with a *P* ≤ 0.05 and a FC ≥ 1.25/1 or FC ≤ 1/1.25 are listed.

| CIEV-Mifepristone vs. Air-Mifepristone | | | |
| --- | --- | --- | --- |
|  | **Gene Symbol** | **Protein Name** | **Log2FC** |
| Downregulated | Nos1 | Nitric oxide synthase, brain | -0.57 |
|  | Tph2 | Tryptophan 5-hydroxylase 2 | -0.55 |
|  | Marcks | Myristoylated alanine-rich C-kinase substrate | -0.52 |
|  | Cd38 | ADP-ribosyl cyclase/cyclic ADP-ribose hydrolase 1 | -0.42 |
|  | Msmo1 | Methylsterol monooxygenase 1 | -0.42 |
|  | Lgals1 | Galectin-1 | -0.42 |
|  | Dhps | Deoxyhypusine synthase | -0.40 |
|  | Calb2 | Calretinin | -0.40 |
|  | Rps14 | 40S ribosomal protein S14 | -0.38 |
|  | Pcsk1n | ProSAAS | -0.37 |
|  | Scn1a | Sodium channel protein type 1 subunit alpha | -0.37 |
|  | Gabrb3 | Gamma-aminobutyric acid receptor subunit beta-3 | -0.36 |
|  | Sec61a1 | Protein transport protein Sec61 subunit alpha isoform 1 | -0.36 |
| Upregulated | Lap3 | Cytosol aminopeptidase | 0.32 |
|  | Prkcb | Protein kinase C beta type | 0.32 |
|  | Glul | Glutamine synthetase | 0.34 |
|  | Mug1 | Murinoglobulin-1 | 0.34 |
|  | Rhoa | Transforming protein RhoA | 0.38 |
|  | Gjc2 | Gap junction gamma-2 protein | 0.39 |
|  | Gpm6a | Neuronal membrane glycoprotein M6-a | 0.42 |
|  | Elp5 | Elongator complex protein 5 | 0.42 |
|  | Ppme1 | Protein phosphatase methylesterase 1 | 0.49 |
|  | S100a1 | Protein S100-A1 | 0.52 |
|  | Adcy5 | Adenylate cyclase type 5 | 0.57 |
|  | Tuba4a | Tubulin alpha-4A chain | 0.58 |
|  | Gng7 | Guanine nucleotide-binding protein G(I)/G(S)/G(O) subunit gamma-7 | 0.58 |
|  | Ca1 | Carbonic anhydrase 1 | 0.64 |
|  | Igg-2a | Ig gamma-2A chain C region | 0.64 |
|  | Hba1 | Hemoglobin subunit alpha-1/2 | 0.66 |
|  | Cox7c | Cytochrome c oxidase subunit 7C, mitochondrial | 0.68 |
|  | Abca3 | Phospholipid-transporting ATPase ABCA3 | 0.75 |
|  | Gnb1 | Guanine nucleotide-binding protein G(I)/G(S)/G(T) subunit beta-1 | 2.16 |
|  | H3-3b | Histone H3.3 | 4.22 |

***Supplemental Table 7*. A comprehensive list of all proteins significantly downregulated or upregulated by CIEV in the context of mifepristone in female Wistar rats.** The gene symbols, protein names, and Log2FC are included in the table. Only those with a *P* ≤ 0.05 and a FC ≥ 1.25/1 or FC ≤ 1/1.25 are listed.

| Air-Mifepristone vs. Air-Placebo | | | |
| --- | --- | --- | --- |
|  | **Gene Symbol** | **Protein Name** | **Log2FC** |
| Upregulated | Scn1a | Sodium channel protein type 1 subunit alpha | 0.35 |
|  | Nos1 | Nitric oxide synthase, brain | 0.38 |
|  | Glrb | Glycine receptor subunit beta | 0.49 |
|  | Ptdss2 | Phosphatidylserine synthase 2 | 0.51 |

***Supplemental Table 8*. A comprehensive list of all proteins significantly downregulated or upregulated by mifepristone in female Wistar rats.** The gene symbols, protein names, and Log2FC are included in the table. Only those with a *P* ≤ 0.05 and a FC ≥ 1.25/1 or FC ≤ 1/1.25 are listed.

| CIEV-Mifepristone vs. CIEV-Placebo | | | |
| --- | --- | --- | --- |
|  | **Gene Symbol** | **Protein Name** | **Log2FC** |
| Downregulated | Cck | Cholecystokinin | -1.06 |
|  | Sst | Somatostatin | -0.57 |
|  | Marcks | Myristoylated alanine-rich C-kinase substrate | -0.41 |
|  | Basp1 | Brain acid soluble protein 1 | -0.41 |
|  | Synpo | Synaptopodin | -0.40 |
|  | Hpcal1 | Hippocalcin-like protein 1 | -0.40 |
|  | Cpe | Carboxypeptidase E | -0.39 |
|  | Scg2 | Secretogranin-2 | -0.39 |
|  | Tubb2b | Tubulin beta-2B chain | -0.39 |
|  | Tmsb4x | Thymosin beta-4 | -0.38 |
|  | Map2 | Microtubule-associated protein 2 | -0.37 |
|  | Clvs2 | Clavesin-2 | -0.35 |
|  | Camk2d | Calcium/calmodulin-dependent protein kinase type II subunit delta | -0.35 |
|  | Calm3 | Calmodulin-3 | -0.34 |
| Upregulated | Folh1 | Glutamate carboxypeptidase 2 | 0.33 |
|  | Rtkn | Rhotekin | 0.34 |
|  | Kcnj10 | ATP-sensitive inward rectifier potassium channel 10 | 0.39 |
|  | Phgdh | D-3-phosphoglycerate dehydrogenase | 0.41 |
|  | Tuba4a | Tubulin alpha-4A chain | 0.44 |
|  | Prkcb | Protein kinase C beta type | 0.46 |
|  | Gpm6a | Neuronal membrane glycoprotein M6-a | 0.47 |
|  | Krt5 | Keratin, type II cytoskeletal 5 | 0.48 |
|  | Mug1 | Murinoglobulin-1 | 0.49 |
|  | Adcy5 | Adenylate cyclase type 5 | 0.50 |
|  | Gpd1 | Glycerol-3-phosphate dehydrogenase [NAD(+)], cytoplasmic | 0.52 |
|  | Cox7c | Cytochrome c oxidase subunit 7C, mitochondrial | 0.52 |
|  | Sirt2 | NAD-dependent protein deacetylase sirtuin-2 | 0.54 |
|  | Gng7 | Guanine nucleotide-binding protein G(I)/G(S)/G(O) subunit gamma-7 | 0.56 |
|  | Glul | Glutamine synthetase | 0.57 |
|  | Myo1d | Unconventional myosin-Id | 0.57 |
|  | Cnp | 2',3'-cyclic-nucleotide 3'-phosphodiesterase | 0.58 |
|  | Ca1 | Carbonic anhydrase 1 | 0.60 |
|  | Ina | Alpha-internexin | 0.60 |
|  | Syt2 | Synaptotagmin-2 | 0.64 |
|  | Hba1 | Hemoglobin subunit alpha-1/2 | 0.65 |
|  | Igg-2a | Ig gamma-2A chain C region | 0.65 |
|  | Alb | Albumin | 0.67 |
|  | GLTP | Glycolipid transfer protein | 0.74 |
|  | Heph | Hephaestin | 0.89 |
|  | Pde10a | cAMP and cAMP-inhibited cGMP 3',5'-cyclic phosphodiesterase 10A | 0.93 |
|  | Plp1 | Myelin proteolipid protein | 0.94 |
|  | Scn4b | Sodium channel subunit beta-4 | 1.55 |

***Supplemental Table 9*. A comprehensive list of all proteins significantly downregulated or upregulated by mifepristone in the context of CIEV in female Wistar rats.** The gene symbols, protein names, and Log2FC are included in the table. Only those with a *P* ≤ 0.05 and a FC ≥ 1.25/1 or FC ≤ 1/1.25 are listed.

| CIEV Effects in the Context of Mifepristone (CIEV-Mifepristone/Air-Mifepristone) | | | | | |
| --- | --- | --- | --- | --- | --- |
| Ingenuity  Canonical Pathways | **-log(*p*-value)** | **Ratio** | **Activation z-score** | **Molecules** | |
| G alpha (z) signaling events | 5.92 | 0.0851 | 2 | ADCY5, GNB1, GNG7, PRKCB | |
| Beta-catenin independent WNT signaling | 4.66 | 0.0412 | 2 | GNB1, GNG7, PRKCB, RHOA | |
| P2Y Purinergic Receptor Signaling Pathway | 4.17 | 0.031 | 2 | ADCY5, GNB1, GNG7, PRKCB | |
| Phospholipase C Signaling | 4.15 | 0.0194 | 2 | ADCY5, GNB1, GNG7, PRKCB, RHOA | |
| Eicosanoid Signaling | 4.07 | 0.0187 | 2.236 | ADCY5, GNB1, GNG7, PRKCB, RHOA | |
| Gαq Signaling | 3.77 | 0.0244 | 2 | GNB1, GNG7, PRKCB, RHOA | |
| GNRH Signaling | 3.59 | 0.0219 | 2 | ADCY5, GNB1, GNG7, PRKCB | |
| IL-8 Signaling | 3.44 | 0.02 | 2 | GNB1, GNG7, PRKCB, RHOA | |
| Role of NFAT in Cardiac Hypertrophy | 3.32 | 0.0185 | 2 | ADCY5, GNB1, GNG7, PRKCB | |
| Orexin Signaling Pathway | 3.23 | 0.0175 | 2 | ADCY5, GNB1, GNG7, PRKCB | |
| Cardiac Hypertrophy Signaling | 3.1 | 0.0162 | 2 | ADCY5, GNB1, GNG7, RHOA | |
| Colorectal Cancer Metastasis Signaling | 3 | 0.0153 | 2 | ADCY5, GNB1, GNG7, RHOA | |
| Oxytocin Signaling Pathway | 2.98 | 0.0151 | 2 | GNB1, GNG7, PRKCB, RHOA | |
| Cardiac Hypertrophy Signaling (Enhanced) | 2.75 | 0.00962 | 2.236 | ADCY5, GNB1, GNG7, PRKCB, RHOA | |
| Molecular Mechanisms of Cancer | 1.94 | 0.00619 | 2.236 | ADCY5, GNB1, GNG7, PRKCB, RHOA | |
| G-Protein Coupled Receptor Signaling | 1.59 | 0.00605 | 2 | ADCY5, GNB1, GNG7, PRKCB | |
| Mifepristone Effects in the Context of CIEV (CIEV-Mifepristone/CIEV-Placebo) | | | | | |
| Ingenuity Canonical Pathways | **-log(*p*-value)** | **Ratio** | **Activation z-score** | **Molecules** |  |
| Docosahexaenoic Acid (DHA) Signaling | 2.69 | 0.0168 | 2 | ADCY5,ALB,PRKCB,SYT2 |  |

***Supplemental Table 10*. Ingenuity canonical pathways significantly altered by CIEV in the context of mifepristone and mifepristone in the context of CIEV in female Wistar rats.** The table includes all the Ingenuity canonical pathways altered, -log(*p*-value), ratio, activation z-score, and the proteins involved in the respective pathway. Only those with a *p-*value ≤ 0.05 and |activation z-score| ≥ 2 are considered as significant changes and included in this table. CIEV and mifepristone alone did not significantly alter any canonical pathways.

***
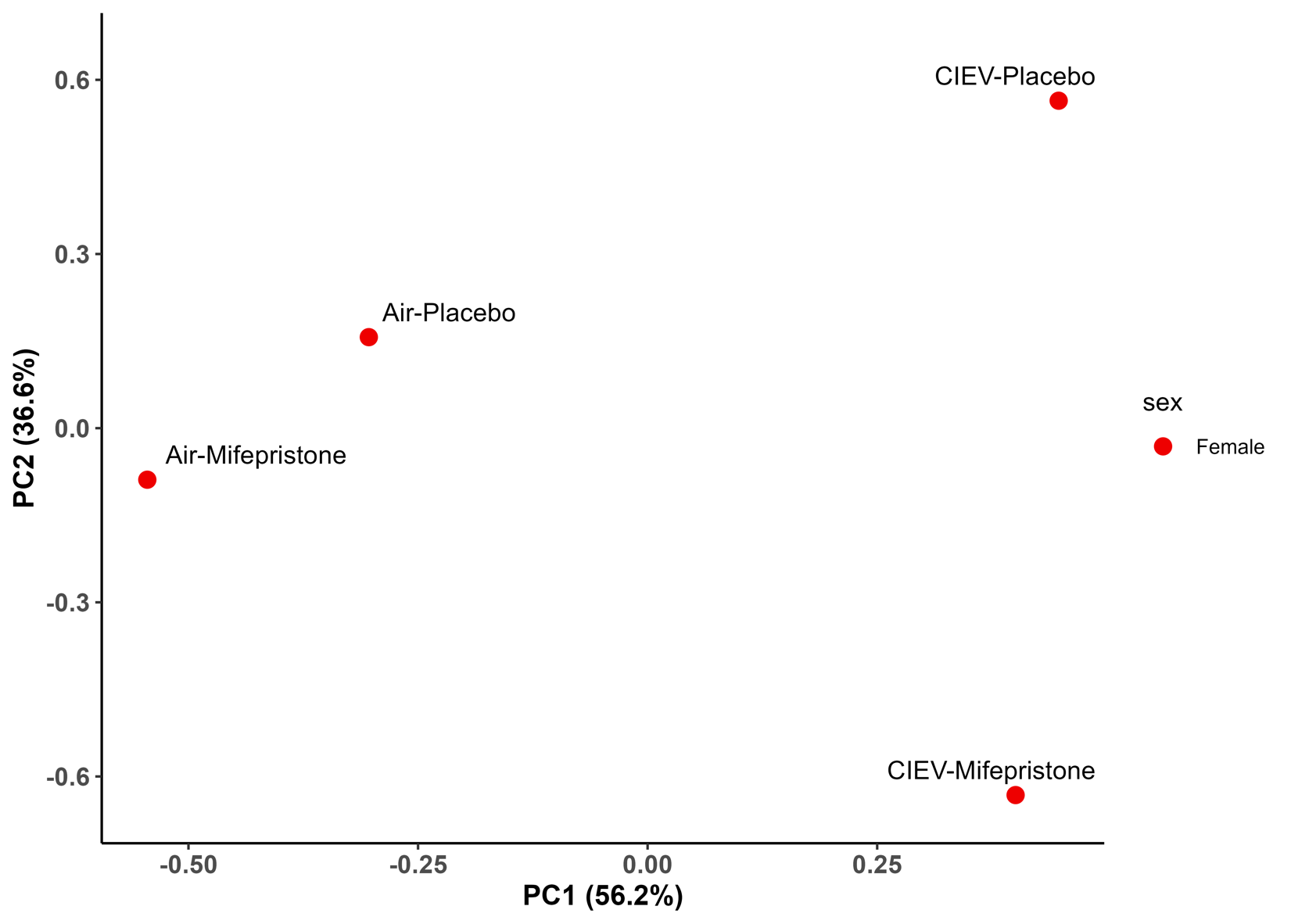
***

***Supplemental Figure 2.* Principal Component Analysis (PCA) score plot of proteomics data of female Wistar rats.** The PCA plot represents >2,600 proteins that were expressed in all groups. Proteomic profiles of CIEV and air animals separate along principal component 1, which explains 56.2% of variability in our data set. Proteomic profiles of mifepristone and placebo animals separate along principal component 2, which explains 36.6% of variability in our data set. Air-Placebo (*n* = 6); Air-Mifepristone (*n* = 6); CIEV-Placebo (*n =* 4); CIEV-Mifepristone (*n* = 4).
